# Variation in chromosome copy number influences the virulence of *Cryptococcus neoformans *and occurs in isolates from AIDS patients

**DOI:** 10.1186/1471-2164-12-526

**Published:** 2011-10-27

**Authors:** Guanggan Hu, Joyce Wang, Jaehyuk Choi, Won Hee Jung, Iris Liu, Anastasia P Litvintseva, Tihana Bicanic, Rajeev Aurora, Thomas G Mitchell, John R Perfect, James W Kronstad

**Affiliations:** 1 The Michael Smith Laboratories, Department of Microbiology and Immunology, and Faculty of Land and Food Systems, University of British Columbia, Vancouver, B.C., V6T 1Z4, Canada; 2 Department of Biotechnology, Chung-Ang University, Anseong-Si, Gyeonggi-Do, 456-756, Republic of Korea; 3 Department of Molecular Genetics and Microbiology, Duke University Medical Center, Durham, North Carolina, USA; 4 Research Centre of Infection and Immunity, St. George's University of London, London, UK; 5 Department of Molecular Microbiology and Immunology, Saint Louis University School of Medicine, St. Louis, Missouri, USA; 6 Department of Medicine, Division of Infectious Diseases and International Health, Duke University Medical Center, Durham, North Carolina, USA

**Keywords:** Comparative genome hybridization, fungal pathogenesis, meningitis

## Abstract

**Background:**

The adaptation of pathogenic fungi to the host environment via large-scale genomic changes is a poorly characterized phenomenon. *Cryptococcus neoformans *is the leading cause of fungal meningoencephalitis in HIV/AIDS patients, and we recently discovered clinical strains of the fungus that are disomic for chromosome 13. Here, we examined the genome plasticity and phenotypes of monosomic and disomic strains, and compared their virulence in a mouse model of cryptococcosis

**Results:**

In an initial set of strains, melanin production was correlated with monosomy at chromosome 13, and disomic variants were less melanized and attenuated for virulence in mice. After growth in culture or passage through mice, subsequent strains were identified that varied in melanin formation and exhibited copy number changes for other chromosomes. The correlation between melanin and disomy at chromosome 13 was observed for some but not all strains. A survey of environmental and clinical isolates maintained in culture revealed few occurrences of disomic chromosomes. However, an examination of isolates that were freshly collected from the cerebrospinal fluid of AIDS patients and minimally cultured provided evidence for infections with multiple strains and copy number variation.

**Conclusions:**

Overall, these results suggest that the genome of *C. neoformans *exhibits a greater degree of plasticity than previously appreciated. Furthermore, the expression of an essential virulence factor and the severity of disease are associated with genome variation. The occurrence of chromosomal variation in isolates from AIDS patients, combined with the observed influence of disomy on virulence, indicates that genome plasticity may have clinical relevance.

## Background

The adaptation of pathogens to the host environment is a critical determinant of the outcome of disease. Well-documented examples include antigenic variation in parasites and fungi to evade adaptive immune responses, viral evasion of immune detection, and phase variation in bacterial pathogens [1-5]. For fungi that attack humans, accumulating evidence indicates that genomic plasticity also contributes to adaptation to the host. For example, surveys of clinical isolates of *Candida albicans *reveal extensive variation in karyotypes and chromosome copy number, and genomic changes have been shown to arise during infection [6-11]. Furthermore, aneuploidy is common in laboratory strains of *C. albicans*, and this trait, along with isochromosome formation, is associated with resistance to azole antifungal drugs [10-15]. Genome-wide variation also occurs during infections caused by *Cryptococcus neoformans*, the leading cause of fungal meningoencephalitis in patients with HIV/AIDS [16]. For example, Fries et al. [17] documented karyotypic changes and gross chromosomal rearrangements or deletions in sequential isolates from individual patients. Subsequent experimental infections in mice also confirmed that karyotypic changes occur during passage in the host [17].

Previously, we employed comparative genome hybridization (CGH) to characterize chromosomal variations among strains of *C. neoformans *that differed in capsular serotypes, molecular subtypes and/or ploidy, including hybrids of different serotype [18]. *C. neoformans *generally exists as a haploid yeast in the environment and patients, although natural hybrid strains are also found and they appear to be diploid or aneuploid [19-21]. Our CGH analysis of three strains of hybrid serotype (AD strains) revealed that these strains were aneuploid with preferential retention of chromosome 1 from the serotype A genome [18]. This study also evaluated four clinical isolates of serotype A and identified two that were disomic for chromosome 13 [18]. Strains of serotype A are responsible for the majority of clinical cases of cryptococcosis [22-24]. The two disomic clinical isolates came from AIDS patients in Argentina (CBS7779) and Australia (WM626). We examined these strains for the three most important virulence factors, formation of the polysaccharide capsule, deposition of melanin in the cell wall, and growth at 37°C. The strains were phenotypically similar to the standard reference strain in capsule production and growth at 37°C but they exhibited reduced formation of melanin [18]. This result suggested that phenotypic differences related to virulence might be correlated with variation in chromosome copy number. More recently, polyploidy has been described as a feature of *C. neoformans *cells in the host [25,26].

Variation in chromosome content may be more common in *C. neoformans *than previously recognized. In addition to the occurrence of disomy in clinical strains [18], Sionov et al. [27] recently found that selection for growth in the presence of elevated levels of the antifungal drug fluconazole resulted in disomy. Specifically, strains of *C. neoformans *exhibited heteroresistance in which a minor subpopulation of cells was present that could tolerate levels of the drug above the normal minimal inhibitory concentration. The resistant subpopulation represented 0.3-0.6% of the starting cells and CGH analysis revealed that the heteroresistant isolates were disomic for chromosome 1, as well as other chromosomes (i.e., 4, 10, 14) depending on the level of resistance. Notably, chromosome 1 carries both *ERG11*, encoding lanosterol-14-α-demethylase, the target of fluconazole, and *AFR1*, encoding an ABC transporter associated with susceptibility to azoles. Subsequent genetic analysis confirmed a role for these genes in the association of disomy with resistance to fluconazole. Sionov et al. [27] proposed that fluconazole exposure may induce chromosome amplification rather than simply select for disomic strains arising by spontaneous chromosomal mis-segregation. The clinical relevance of chromosomal aberrations is further supported by other recent observations. As mentioned above, changes in genome copy number resulting in polyploidy are observed for so-called giant cells of *C. neoformans *arising during pulmonary infection [25,26]. In addition, Desnos-Ollivier et al. [28] discovered that ~18% of patients have mixed infections with strains of different mating type, serotype, genotype and ploidy.

In the current study, we extended our analysis of disomy in *C. neoformans *by testing the hypothesis that the copy number of chromosomes affects the expression of the virulence trait melanin and the severity of disease in experimentally infected mice. The results confirmed that disomy at chromosome 13 is correlated with both loss of melanin formation and reduced murine virulence. However, we also discovered copy number changes for other chromosomes in strains with altered melanin formation, and the correlation with chromosome 13 was not always observed. The additional changes in chromosome copy number were detected by screening strains that were passaged in culture or through mice. The discovery of disomy in a clinical isolate and its influence on virulence, suggested that disomy might arise during human infections. We therefore screened a collection of clinical and environmental strains, as well as isolates obtained directly from the cerebrospinal fluid of HIV/AIDS patients, and we found additional examples of variation in chromosome copy number. Overall, this study suggests that variation in the expression of virulence traits among strains and their ability to adapt to the mammalian host environment are influenced by changes in chromosome copy number.

## Results

### Disomy at chromosome 13 of *C. neoformans *is correlated with reduced melanin formation

**Figure 1:**
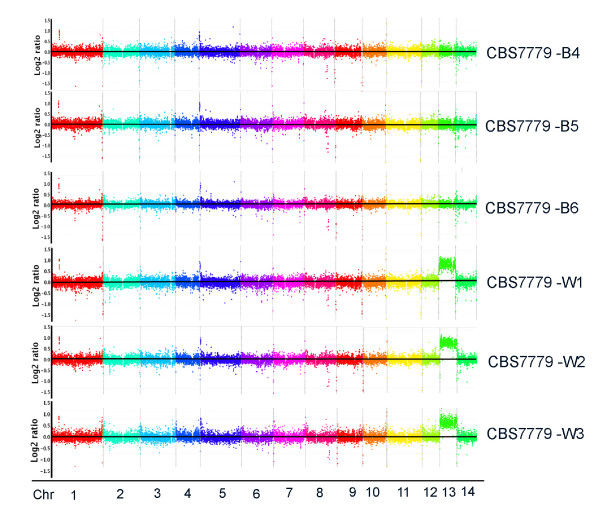
**Comparative genome hybridization reveals disomy for chr 13 in melanin variants**. A custom CGH array (Roche-NimbleGen) for the reference genome of strain H99 was used to examine the black (CBS7779-B4, CBS7779-B5 and CBS7779-B6) and white (CBS7779-W1, CBS7779-W2 and CBS7779-W3). The x-axis represents the position of probes arranged in the order of their chromosomal location. The y-axis denotes the relative hybridization signal intensity that was plotted as the log2 value. Each spot represented a running average value of hybridizations for a genomic segment of 1,300 bps across the chromosomes. All three white strains showed disomy on chromosome 13.

In comparison to the serotype A strain H99, the reference strain that is commonly used to investigate the virulence of *C. neoformans*, two clinical isolates, CBS7779 and WM626, were reported to have significantly diminished production of the major virulence factor melanin [18]. Since both strains were shown by comparative genome hybridization (CGH) to be disomic for chromosome 13 (chr 13), we hypothesized that their reduction in melanin formation was due to chromosomal variation such as disomy. We focused our subsequent work on CBS7779 because this strain was similar in virulence to strain H99, and strain WM626 was attenuated for virulence (see below). To examine the relationship between melanin formation and disomy, we initially screened strain CBS7779 for variants that showed a more pronounced defect in melanin production (hereafter called white variants). These were readily identified along with black variants that formed more highly melanized colonies. This screen yielded 12 black isolates (designated B1-B12), and 12 white isolates (W1-W12) for further analysis. These isolates generally maintained their melanin phenotypes upon repeated streaking on L-DOPA medium. Each of the 12 black and 12 white melanin variants were first examined by quantitative PCR using primers for genes at three positions along chr 13 (CNN00820, CNN01890 and CNN02400) to determine the copy number of the chromosome. Based on the genome sequences of serotype A and D strains [29,30], the actin gene on chr 1 (CNA04650) and a gene on chr 4 (CND00707) were employed as a single copy controls. In addition, the *SMG1 *gene on chr 4 was used as a control because Fraser et al. [31] showed that this gene was duplicated in the serotype D strain JEC21, but not in strain H99. The PCR analysis revealed that each of the 12 black variants was monosomic at chr 13 and chr 4. All 12 of the white variants were monsomic at chr 4 but nine were disomic and three were trisomic at chr 13 (Table 1). These analyses were repeated several times on independent DNA samples with identical results. The PCR results were also confirmed and extended by CGH analysis on independent DNA samples that revealed monosomy for chr 13 in the three black variants (B4-6) and disomy in the three white variants (W1-W3) (Figure 1).

**Table 1:** Analysis of gene copy number in CBS7779 variants relative to strain H99.

Strain	SMG1 (chr 4)	CNN00820 (chr 13)	CND00707 (chr 4)
H99	1	1	1
JEC21	2.33	1.07	1.22
CBS7779 B1	1.1	1.09	ND
CBS7779 B2	1.08	0.95	ND
CBS7779 B3	1.16	1.08	ND
CBS7779 B4	1.1	1.03	1.08
CBS7779 B5	1.24	0.99	1.28
CBS7779 B6	1.16	1.01	1.37
CBS7779 B7	1.27	0.68	ND
CBS7779 B8	1.38	1.34	ND
CBS7779 B9	1.43	1.01	ND
CBS7779 B10	1.38	0.74	ND
CBS7779 B11	1.26	1.15	ND
CBS7779 B12	1.36	0.99	ND
CBS7779 W1	1.3	1.84	ND
CBS7779 W2	1.16	2.01	ND
CBS7779 W3	1.1	1.97	ND
CBS7779 W4	1.23	2.23	1.36
CBS7779 W5	1.11	1.85	1.24
CBS7779 W6	0.96	1.78	1.19
CBS7779 W7	1.29	1.8	ND
CBS7779 W8	1.13	2.66	ND
CBS7779 W9	1.25	2.89	ND
CBS7779 W10	1.15	2.01	ND
CBS7779 W11	1.15	2.87	ND
CBS7779 W12	1.13	3.1	ND

Quantitative PCR was used to examine copy number (2^-ΔΔCt ^method).

ND, not determined

**Figure 2:**
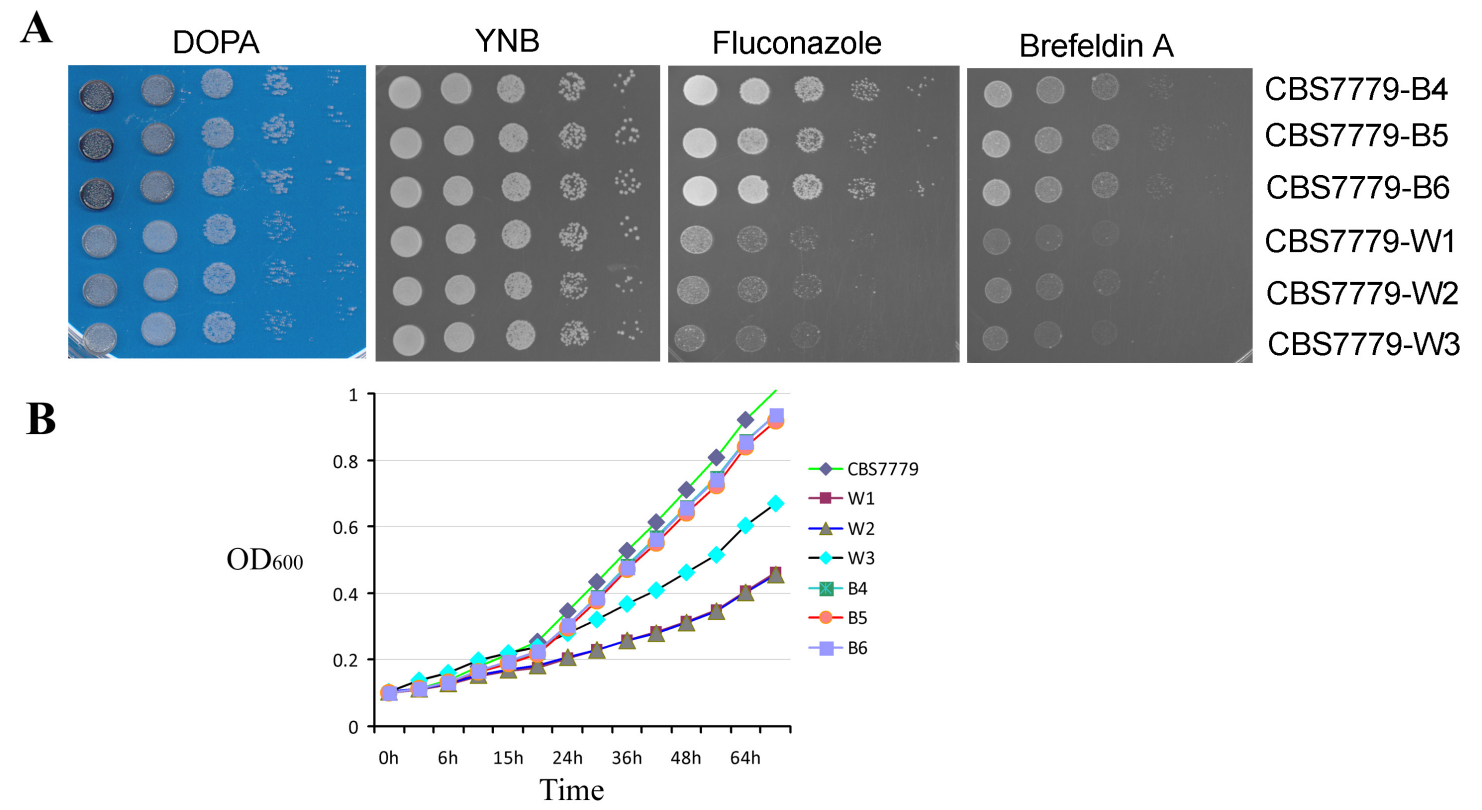
**Melanin variants show differences in growth as well as sensitivity to fluconazole and brefeldin A**. (A) Dilutions of the melanin variants were spotted on the media indicated above the panels with YNB medium as the control for growth in the presence of fluconazole (5 μg/ml) and brefeldin A (20 μg/ml). Growth on the L-DOPA plate demonstrates the difference in melanin production by the strains. The plates were incubated for 2 days at 30°C before being photographed. (B) Growth of the CBS7779 strain and three black and three white melanin variants was measured in a TECAN plate reader. The assay was repeated three times with similar results.

In addition to a correlation between reduced melanization and disomy at chr 13, we also found that white strains were more susceptible to the antifungal drug fluconazole, and to the trafficking inhibitors brefeldin A (BFA) and monensin (Figure 2A and data not shown). The latter inhibitors are relevant to virulence because we previously showed that they inhibit the secretory pathway needed for capsule formation [32]. In addition, several secreted enzymes contribute to virulence in *C. neoformans *including phospholipase B, urease and laccase [33]. The black and white variants exhibited no differences in capsule formation or their responses to oxidative and nitrosative stress (data not shown). However, the white strains did display slower growth in liquid culture (Figure 2B). Overall, these results established a correlation between disomy at chr 13 and phenotypic traits with potential relevance to virulence and clinical outcome, i.e., melanin formation, secretion and susceptibility to fluconazole.

### Disomic, non-melanized strains have reduced virulence

**Figure 3:**
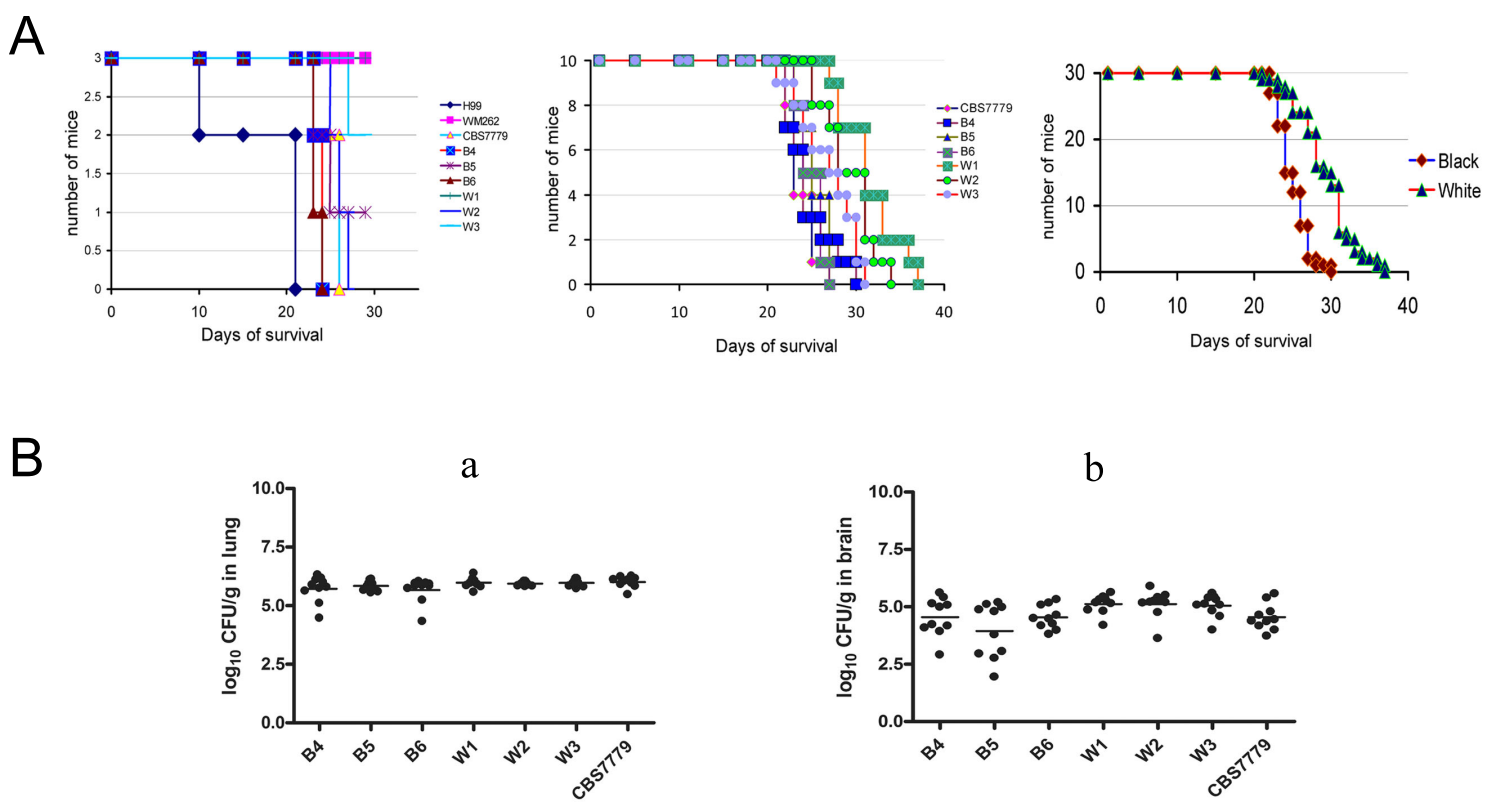
**Monsomic and disomic melanin variants differ in their virulence in a mouse inhalation model but show similar fungal loads in brain and lung tissue**. (A) Three female BALB/c mice were inoculated intranasally with each of the strains indicated, and the survival of the mice was monitored (left graph). The strain H99 was the most virulent, WM626 did not cause illness during the experiment, and the white (disomy) variants showed reduced virulence compared with CBS7779 and the black variants. Ten female BALB/c mice were also challenged by intranasal inoculation with cells of either the CBS7779 strain or the black or white variants. The survival of the mice was monitored (center graph). The white (disomy) strains showed a difference in virulence from the black strains and from the parental CBS7779 strain. The data on the virulence of the white and black strains was combined, and the white strains showed less virulence than the black strains by the log rank test (P < 0.001) (right graph). (B) Examination of the fungal load in lung (a) and brain (b) tissue from the mice infected in (A) with the white or black melanin variants, or with the original CBS7779 strain. The organs from each of the ten mice per inoculated strain were harvested and analyzed for fungal burden. Serial dilutions of the homogenates were plated on Sabouraud dextrose agar plates, and colony-forming units were counted after incubation for 48 h at 30°C. Each dot indicates the result for a single mouse.

The white and black variants with defined chromosomal complements provided the opportunity to determine the effect, if any, of variation in chromosome copy number on virulence in a mouse inhalation model of cryptococcosis. Cells from the same cultures that were employed to isolate DNA for the CGH analysis of the three white (W1-3) and black (B4-6) variants were also used as inoculum in the virulence assays. Initially, we performed a pilot virulence test by inoculating three mice with each of the three black isolates and the three white isolates. This study also included strain H99 because of its well-characterized and high level of murine virulence, as well as the original CBS7779 and WM626 strains. The results indicated that CBS7779 was similar in virulence to strain H99, WM626 was attenuated for virulence, and the white (disomic) variants were less virulent than the black (monosomic) variants (Figure 3A). These results were substantiated in a larger study that employed 10 mice for each variant. As shown in Figure 3A, the white variants again showed reduced virulence (i.e., prolonged survival) compared with the black variants.

**Figure 4:**
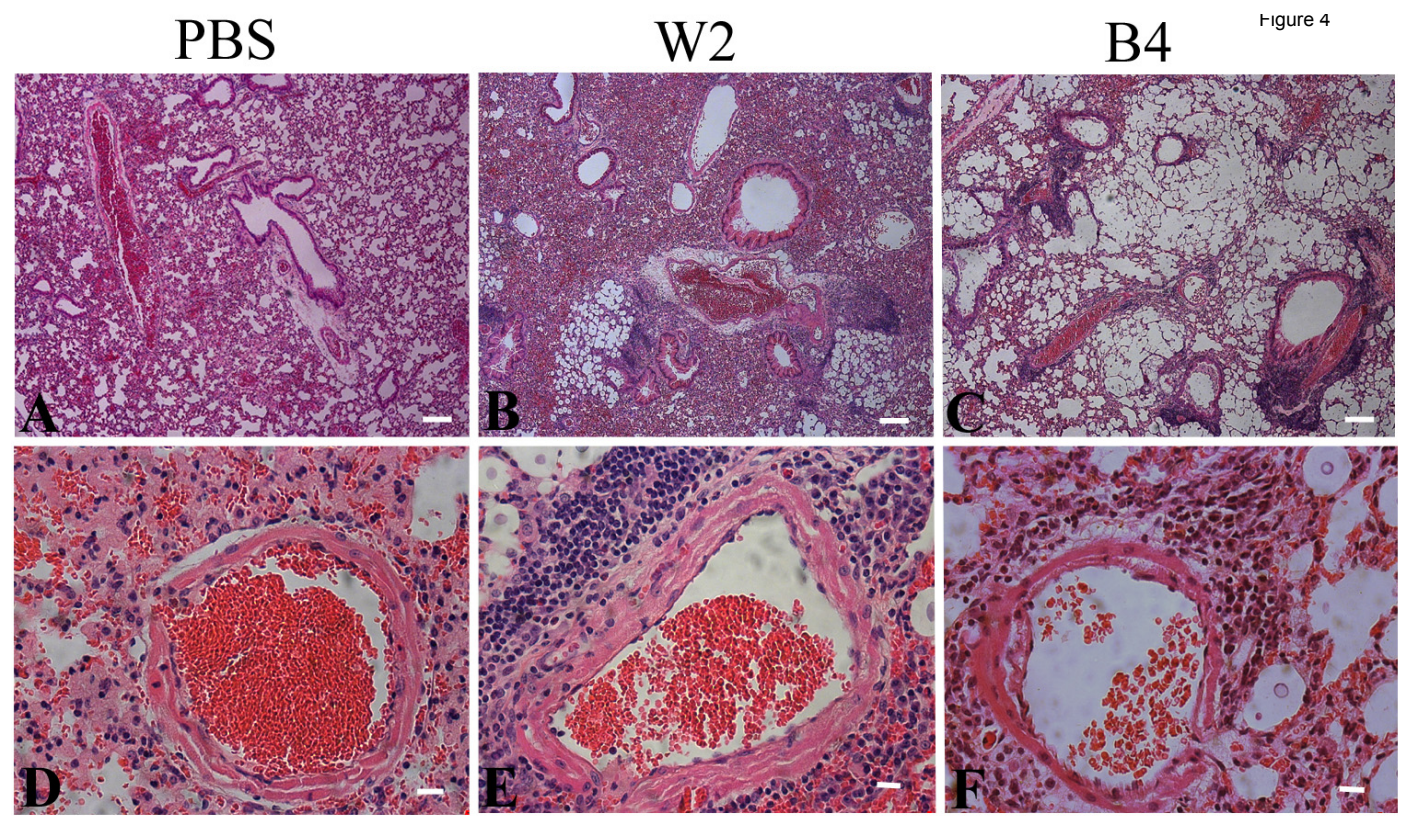
**Histopathology of lung tissue infected with melanin variants**. Thin sections of pulmonary tissue from the uninfected mice (A, D, PBS control), or mice infected with either CBS7779-W2 (B, E) or CBS7779-B4 (C, F) (21 days post-inoculation, dpi) were stained with hematoxylin and eosin (H&E). The inflammatory response at 21 days post infection (dpi) was similar to that on 14 dpi (data not shown). Compared to the control (A), the gas exchange area in CBS7779-W2 infected lungs was largely reduced due to tissue thickening (B). Lung tissues were severely damaged in CBS7779-B4 infected mice (C). No marked inflammatory response was observed in control (D), while the infection by either CBS7779-W2 (E) or CBS7779-B4 (F) caused similar levels of peribronchial inflammation. Blood vessels within the lungs infected with CBS7779-W2 (E) were surrounded by more inflammatory infiltrates than those within CBS7779-B4 infected lungs (F). Scale bar: 100 μm in A, B, C; 10 μm in D, E, F).

Infections with the black and white variants produced similar fungal burdens in the lungs and the brains of infected mice, although greater variation between mice was observed for the black variants in the brain (Figure 3B). Considering that white variants grew more slowly in culture and the black variants were more lethal, their comparable fungal loads in mice were unexpected. These results suggested that other properties besides fungal proliferation were contributing to disease, and we therefore examined lung and brain tissue by histopathology to more closely compare the infections. As shown in Figure 4, lungs infected with the black strain (CBS7779-B4) exhibited more extensive airway remodeling compared with the lungs infected with the white strain (CBS7779-W2), which showed greater damage to the lung parenchymal as indicated by thickened tissue and reduce air space. These differences in pulmonary histopathology were more pronounced at days 14 and 21 than at day 7 (Figure 4 and data not shown). The infections with both strains resulted in similar levels of perivascular and peribronchial inflammation (Figure 4). Overall, the distinct virulence properties of the black and white strains are associated with differences in the type of damage observed in infected lung tissue.

### Disomy at chromosome 13 influences gene expression

**Figure 5:**
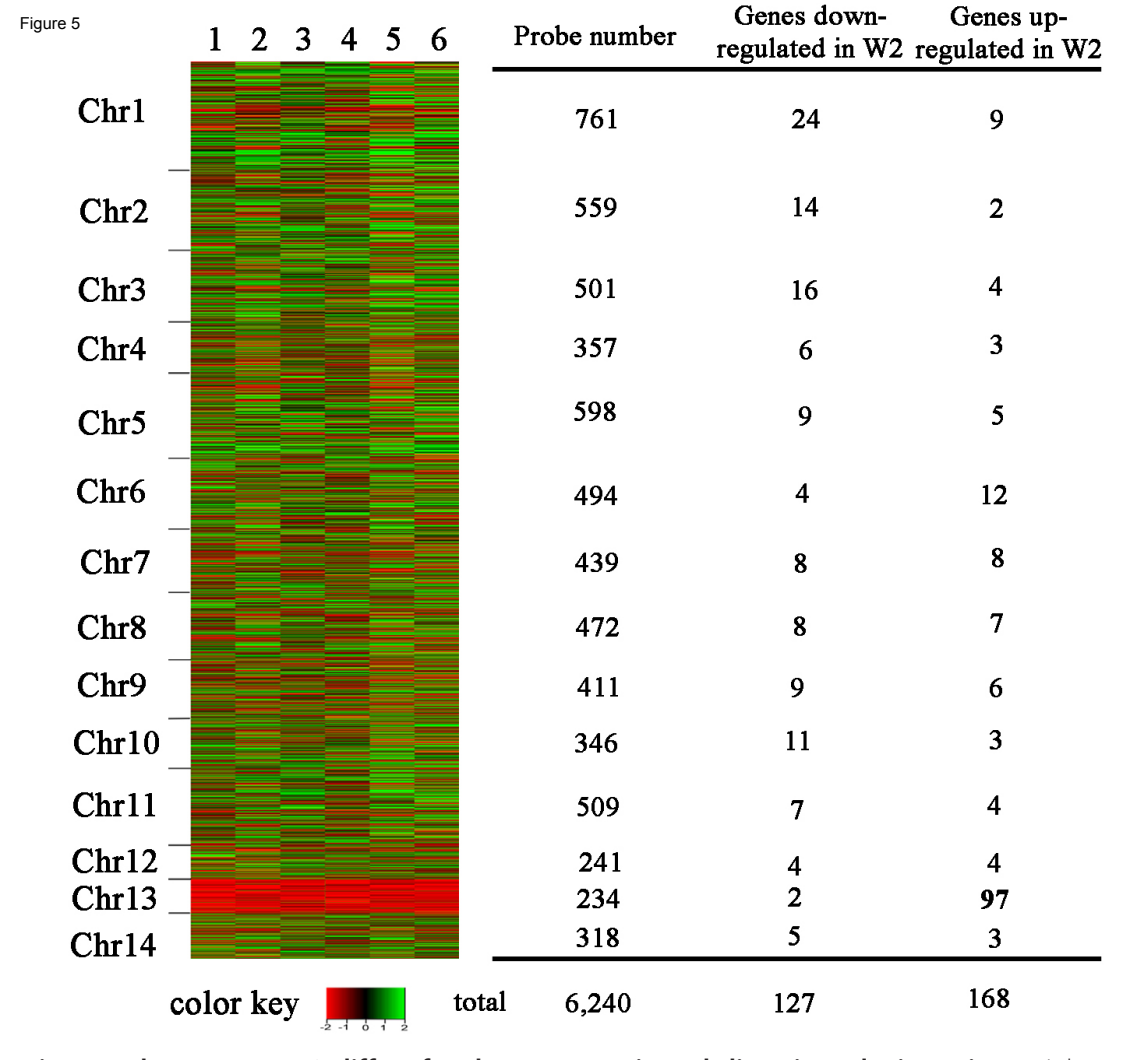
**Gene expression on chromosome 13 differs for the monosomic and disomic melanin variants**. A heat map is shown for the profiles of gene expression of CBS7779-B4 compared to CBS7779-W2 as detected by microarray anlaysis. A total of six arrays including three biological repeats and dye-swap sets were performed. Each column (lane 1 to 6) represents a microarray experiment and each row represents the expression of a gene on the array arranged by its chromosomal position. The relative expression levels are depicted as shown by the color bar. A summary of the numbers of differentially regulated genes for each chromosome in the disomic strain CBS7779-W2, compared to those in CBS7779-B4, is given on the right.

A microarray comparison of the transcriptomes of the black and white isolates was performed to determine whether disomy at chr 13 influences gene expression (Figure 5). For this study, CBS7779 isolates W2 and B4 were grown in liquid L-DOPA medium for six hours and RNA was prepared for hybridization. The analysis revealed that 127 genes were down regulated and 168 were up regulated by two-fold or more in the disomic W2 strain relative to the B4 strain (Additional files 1 and 2). Only two of the down-regulated genes in W2 were on chr 13, compared with 97 of the up-regulated genes. Chr 13 is 756,744 bp in length and contains ~234 annotated genes (see Materials and Methods). Thus, the array data indicated that 41% of the chr 13 genes were up regulated two-fold or more in association with disomy for this chromosome. We also examined the expression of genes on chr 13 (or other chromosomes) that might influence melanin formation and identified the *CCC2 *gene (CNAG_06415) as a potential candidate. Walton et al. [34] found that loss of *CCC2 *results in a melanin defect, and this gene encodes a transporter that may load copper onto the laccase enzyme that catalyzes the polymerization of melanin. However, the expression of *CCC2 *was not substantially different between the B4 and W2 strains (~1.5X higher in the B4 strain) and additional work will be needed to test whether this subtle difference influences melanization.

### Copy number variation at other chromosomes also correlates with melanin formation

**Figure 6:**
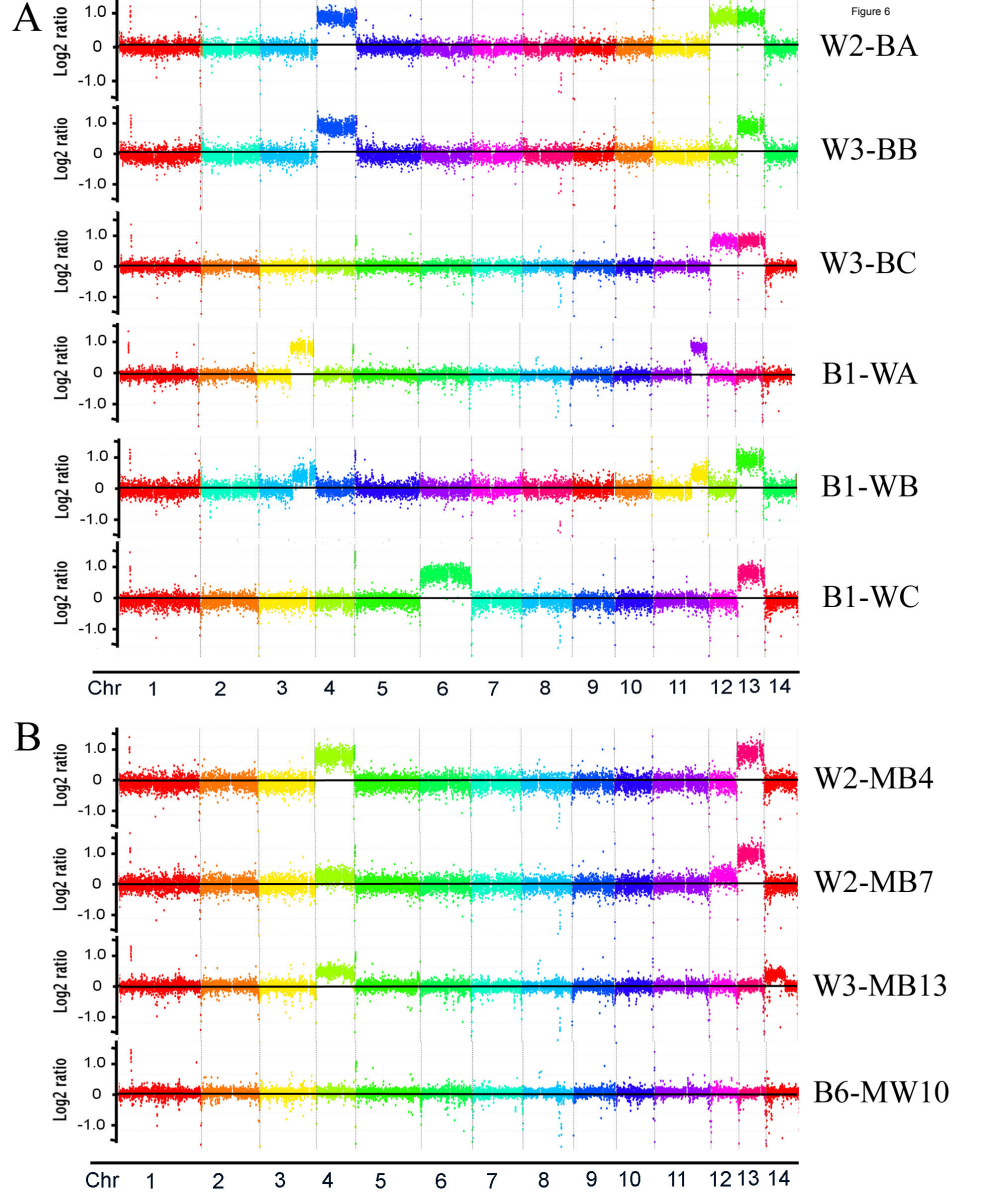
**Copy number variation occurs at the other chromosomes in strains derived from CBS7779**. Genome hybridization to compare "second generation" black strains (A) and white strains (B) to the genome of the reference strain H99. The CGH data for the parental strains CBS7779-W2, -W3 and -B1 are shown in Figure 1 and Additional file 4. The chromosome color labels for the hybridizations with the strains W2-BA, W3-BB and B1-WB come from a separate experiment and are therefore different from the labels used for the other strains.

To further examine the association between chromosome copy number and melanization, we plated the white strains CBS7779-W2 and CBS7779-W3 on L-DOPA medium and screened for "second generation" black variants under the same conditions employed earlier for the parental strain CBS7779. These variants were readily detected and we used CGH to examine the complement of chromosomes in randomly selected colonies (Figure 6A, Table 2). All four of the black variants tested maintained the initial disomy at chr 13 that was present in the starting strains, but they were also disomic for chr 4 (variant W3-BB), chr 12 (W2-BB and W3-BC) or both chr4 and 12 (W2-BA). A subsequent comparison of the transcriptomes of strains W2-BA and B4 also revealed elevated transcription of many of the genes on chr 4, 12 and 13 (Additional file 3). The results for chr 4 and chr 13 in these strains were confirmed by qPCR (data not shown). With regard to melanin formation in the strains, we hypothesize that disomy at chr 4 and/or chr 12 may have compensated for disomy at chr 13. The examination of a random white colony from the starting strain W2 revealed that disomy at chr 13 was maintained and that additional changes occurred for segments of chr 3, 5, 12 and 14 (Table 2, Additional file 4).

**Table 2:** Summary of chromosome changes in second-generation black and white variants collected in culture and from infected mice.

Starting strain/color	Aberrant chromosome	2^nd ^Generation strain^a^/pigment	Aberrant chromosome(s)
**Strains collected in culture**			
**White to black**			
W2/white	Chr 13	W2-BA/black	Chr 13, 4, 12
W2/white	Chr 13	W2-BB/black	Chr 13, 12^b^
W3/white	Chr 13	W3-BB/black	Chr 13, 4
W3/white	Chr 13	W3-BC/black	Chr 13, 12
**White to white**			
W2/white	Chr 13	W2-WB/white	Chr 13 (3, 5, 12, 14)^b^
**Black to white**			
B1/black	none	B1-WA/white	Chr 3^b^, 11^b^
B1/black	none	B1-WB/white	Chr 13 (3, 5, 11)^b^
B1/black	none	B1-WC/white	Chr 13, 5^b^, 6

**Strains collected from mice**			
**White to black**			
W2/white	Chr 13	W2-MB3/black	Chr 13, 4
W2/white	Chr 13	W2-MB4/black	Chr 13, 4
W2/white	Chr 13	W2-MB5/black	none
W2/white	Chr 13	W2-MB7/black	Chr 13, 4, 12
W2/white	Chr 13	W2-MB8/black	Chr 13, 4
W3/white	Chr 13	W3-MB13/black	Chr 4, 14^b^
W3/white	Chr 13	W3-MB16/black	none
**White to white**			
W2/white	Chr 13	W2-MW1/white	Chr 13
W2/white	Chr 13	W2-MW2/white	Chr 13
W3/white	Chr 13	W3-MW6/white	Chr 13
**Black to white**			
B6/black	none	B6-MW10/white	none
B6/black	none	B6-MB20/white	none

^a^The second generation strains are named after the starting strain followed by the color (B = black and W = white) and a letter or number designation. In the case of strains collected from mice, an M is added before the letter indicating color.

^b^These chromosomes show segmental changes. Note that some chromosomes show an elevated copy number but not full disomy perhaps indicating variability among cells in the population.

Three second-generation white variants (B1-WA, B1-WB, B1-WC) from the monosomic black strain CBS7779-B1 were also examined and found to show changes in additional chromosomes (Figure 6A, Table 2). By CGH, (i) strain B1-WA had an increase in the number of copies of segments of chr 3 and chr 11, (ii) B1-WB was disomic for chr13 and showed duplications of segments of chr 3, chr 5 and chr 11, and (iii) strain B1-WC had duplications of chr 13, chr 6 and a segment of chr 5. These results and those described below indicated that changes in copy number occurred for several chromosomes and involved partial or complete chromosome duplication. In some cases, changes in melanization correlated with changes in specific chromosomes (i.e., chr 4, 12, and 13). However, the observed variation for part or all of four additional chromosomes (chr 3, 5, 11, 14) suggests that genome plasticity and/or other factors, such as background mutations, may also influence melanization. Overall, these results indicate that changes in chromosome copy number readily occur in the CBS7779 variants.

### Chromosome copy number variation is detected after passage in mice

We next tested whether passage through mice influenced the transitions from the monosomic and disomic states. Initially, we examined the transition from disomy to monosomy by examining colonies recovered from the mice employed in the virulence assay with the three white strains CBS7779-W1, -W2 and -W3 (Figure 3A). To screen for melanin formation as an indicator of disomy, we determined the proportion of black variants in the starting inoculum of each strain by plating ~30,000 cells of each strain on L-DOPA medium. The W1 inoculum yielded nine black colonies (0.03%) and both W2 and W3 produced six (0.02%). We then determined the fungal burden in the lungs and brains of each mouse (Figure 3B) and screened ~50,000 colonies from each inoculated group (W1-W3) for black colonies. We found the following average percentages of black variants in brain (B) and lung (L) tissue: W1 (B: 0.08%, L: 0.32%), W2 (B: 0.06%, L: 0.22%), W3 (B: 0.05%, L: 0.30%). It appeared from these data that the percentage of black colonies recovered from the brain was lower than that recovered from the lung. This result may indicate a tissue-specific influence on the appearance of melanin variants.

From mice inoculated with the three white strains, we randomly selected 10 white and 14 black colonies and analyzed their chromosome copy numbers. Analysis by qPCR revealed that nine of the white isolates and seven of the black isolates from mice were disomic for chr 13 (data not shown). As with the *in vitro *analysis, this finding indicates that some strains can recover the ability to form melanin while still maintaining disomy at chr13. Subsequent CGH analysis of seven black colonies revealed that two had become monosomic at all chromosomes, three had retained the disomy at chr 13 and gained disomy for chr 4, one had lost disomy at chr 13 but gained disomy at chr 4 with a segmental change in chr 14, and one retained disomy at chr 13 with a elevated copy number of both chr 4 and 12 (Figure 6B, Table 2 and Additional file 5). These results are similar to those obtained with the second-generation black colonies from cultures (described above), where disomy at chr 4 and/or chr 12 correlated with the restoration of melanin formation in strains originally disomic for chr 13. Three white colonies recovered from mice infected with white strains maintained disomy at chr 13 (Figure 6B, Table 2, Additional file 5).

We also examined whether disomy at chr 13 arises in the host by inoculating each of six mice with ~ 1000 cells of the black strain B4. The starting inoculum did not contain white variants among ~30,000 colonies screened prior to inoculation of mice. Mice were sacrificed 24 days after infection and a screen of 2933 colonies from homogenized brain tissue did not reveal any white colonies. However, a screen of ~50,000 colonies from lung tissue yielded 27 white colonies, and subsequent qPCR analysis of 13 of these white isolates revealed that five were disomic for chr 13 (data not shown). In contrast, six randomly selected black colonies that arose from lung tissue were found to be monosomic for chr 13. Although we have not examined other chromosomes, this analysis confirmed that the transition from monosomy to disomy at chr 13 occurs during infection, as well as *in vitro*. However, it was also clear that other mechanisms besides disomy at chr 13, such as the accumulation of background mutations, may result in reduced melanin production.

### Disomy at chromosome 13 occurs in a laboratory strain but does not correlate with melanin formation

**Figure 7:**
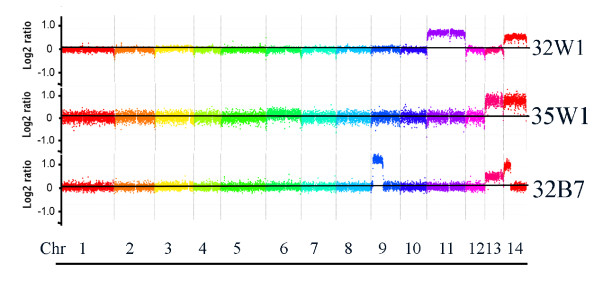
**Targeted selection for disomy at chromosome 13 yields strains with changes in several chromosomes**. The CGH data are shown for three of the six strains selected for neomycin resistance in a derivative of the H99 strain tagged on chr 13 with the neomycin resistance gene. Elevated copy numbers are evident for chr 13 in the white strain 35W1 but also for the black strain 32B7. Copy number changes are also evident for chr 9, 11 and 14 in some of the strains.

The link between disomy at chr 13 and melanin formation was discovered in strain CBS7779 during a survey of genome variation in serotype A strains, and it is possible that strain CBS7779 is particularly prone to changes in chromosome copy number. To determine whether these results are strain-specific or a general property of *C. neoformans*, we investigated whether disomy readily occurs at chr 13 in a laboratory strain. We were also interested in testing whether specific chromosomes could be amplified through selection. We therefore tagged chr 13 in laboratory strain H99 by insertion of a neomycin resistance marker and then plated tagged strains on medium containing neomycin at 10X the concentration generally employed for selection of DNA transformants (2 mg/ml). Four tagged transformants (#s 32, 33, 34 and 35) were plated on L-DOPA medium containing neomycin at 10X the inhibitory concentration (10 × 10^6 ^cells per plate). After five days of incubation, approximately 10 colonies were observed on each plate. White and black colonies were identified on L-DOPA medium with neomycin and the isolates were characterized for capsule formation and cell morphology (Additional file 6). Two of the white variants showed a larger cell size and two showed larger capsules, which indicated phenotypic changes beyond melanin formation. Subsequent CGH analysis of four white and two black isolates revealed that only two strains, one white (35W1) and one black (32B7), had become disomic at chr 13, and both of these strains also had changes at chr 14. One white strain (32W1) had changes at chr 11 and chr 14, and three strains (two white and one black) had no chromosome changes. The CGH results for the three strains with chromosomal changes are shown in Figure 7. Overall, these results indicate that elevated copy number at chr 13 could be achieved in a laboratory strain through tagging a specific chromosome with a selectable marker. However, this approach was only partially effective, and in one strain, an elevated chr 13 copy number did not correlate with reduced melanin formation.

### Disomy is relatively rare in a collection of clinical and environmental strains

**Figure 8:**
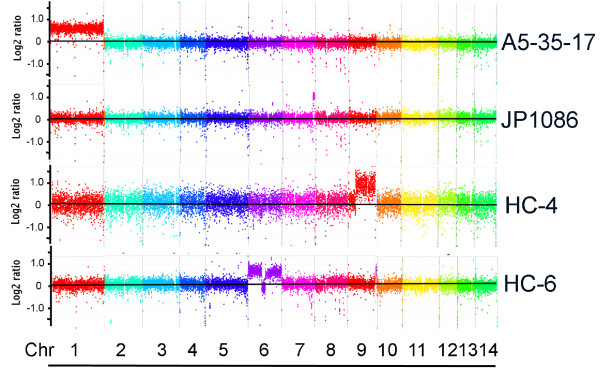
**Chromosome copy number variation or segmental duplications are present in environmental and clinical strains**. CGH analysis revealed that strains A5-35-17 and JP1086 show copy number variation at chr 1 (whole chromosome) and 7 (segmental duplication), respectively, among 19 clinical and environmental isolates. The analysis of isolates from the AIDS patients revealed two, HC-4 and HC-6, with segmental duplications at chr 9 and chr 6, respectively. The "fuzzy" signal intensity for each chromosome in the HC-4 hybridization indicates that the genome of this strain is divergent from the genome of the reference strain H99. This pattern is seen with strains that differ in molecular subtype (VNII or VNB) from the VNI type of H99 [18]. The HC-6 strain was found to be diploid by FACS (Additional file 10) and this suggests that the strain may contain one intact copy of chr 6 and one copy with a central deletion.

Our initial examination of four serotype A strains revealed two (CBS7779 and WM626) that were disomic at chr 13 [18]. To more widely assess the frequency of disomy, we used CGH to survey 19 additional clinical and environmental serotype A strains of different global origins (Additional files 7 and 8). Only two of the isolates showed chromosome copy number changes (Figure 8). Specifically, strain A5-35-17 appeared to have an elevated copy number for chr 1, and strain JP1086 contained an amplified region on chr 7. Both have the A5/M5 genotype as defined by multilocus sequence typing (MLST) and amplified fragment length polymorphism (AFLP) analyses [23,24]. The CGH analysis did reveal that strains A7-35-23 and C45 had a greater overall level of sequence divergence from H99; this result is consistent with the fact that these strains have a VNII molecular type compared with the VNI subtype of strain H99 [24]. We previously demonstrated the ability of CGH to distinguish the three molecular types of serotype A strains, VNI, VNII and VNB [18]. These isolates have been stored and subcultured since the time of their isolation from patients or the environment, and this prolonged period of *in vitro *growth in complete medium may have selected for strains with a haploid complement of chromosomes.

### Chromosome copy number variation is present in isolates from AIDS patients

We next examined whether chromosome copy number variation could be detected in isolates freshly collected from the cerebrospinal fluid of AIDS patients. Multiple colonies were obtained upon plating cerebral spinal fluid from 13 patients and individual colonies from each specimen were used to prepared DNA for CGH. No differences in chromosome copy number were observed for the isolates from 11 of the patients (RCT23, RCT31, HC2, HC3, HC5, RCT17, RCT50, RCT52, RCT55 (day 1), RCT55 (day 4) and RCT60 (data not shown, additional file 9). However, three different colonies from the patient HC2 had divergent patterns of hybridization on the CGH array containing the H99 genome, suggesting sequence heterogeneity. Subsequent testing revealed that these isolates were D serotype or molecular type VNIV (data not shown). It is known that the genomes of A and D strains exhibit sequence divergence of 5-10% [18,29,30]. A divergent hybridization pattern was also noted for two colonies of patient HC3 (HC-3a and HC-3b), but a third colony (HC-3c) showed a tighter hybridization pattern; this result also suggests that a mixed infection of both serotype A and D strains was present in HC3 (Additional file 9). This observation is reminiscent of the finding by Desnos-Ollivier et al. [28] that mixed infections are relatively common (see Discussion below). The colonies obtained from patient HC4 also indicated a mixed infection because one colony showed copy number elevation for part of chr 9 (indicating a segmental duplication, Figure 8), and another colony showed no copy number variation. Interestingly, a copy number change at chr 6 was present in all three of the isolates tested from patient HC6 (Figure 8). In these isolates, it appeared that the chromosome was duplicated but that one copy had a deleted region near the center of the chromosome. It is possible that duplication of the deleted region is not tolerated. FACS analysis indicated that the HC-6 isolates were diploid (Additional file 10). Overall, these results indicated that variation in chromosomal copy number was present in isolates from patients with HIV/AIDS, and provided additional evidence for mixed infections.

## Discussion

Emerging evidence supports the view that the genome of *C. neoformans *exhibits plasticity beyond the haploid euploidy commonly observed in most clinical and environmental isolates [18,25-28,33]. As demonstrated here, genomic variation such as aneuploidy appears to have a major impact on the virulence of *C. neoformans *in a mammalian host. However, detailed investigations are needed to link specific changes to pathogenesis and the clinical outcome of cryptococcosis. In our study, we made the key observation that the black (monosomic) and white (disomic) variants of CBS7779 differed in their virulence in mice. This difference may be attributed to melanin formation because mutants defective in melanin are known to have reduced virulence and impaired dissemination to the brain [34-38]. However, the numbers of yeast cells in lung and brain tissue were similar for the black and white strains. These observations argue against melanin as the sole reason for the difference in virulence. Rather, it appears that melanin formation, although particularly sensitive to variation in chromosome copy number in CBS7779, may be just one of a number of traits affected by chromosomal changes. That is, aneuploidy in general may impair virulence through a number of mechanisms. For example, in other fungi, such as *S. cerevisiae*, aneuploidy results in a decreased growth rate and an imbalance in gene expression [39,40]. For *C. neoformans*, we found that white strains grew more slowly in culture but their proliferation in the host was not impaired. Thus our *in vitro *growth conditions did not reflect proliferation *in vivo*. Our histopathological observations also suggested other contributors to the observed difference in virulence. For example, it appeared that infections with black strains resulted in more airway remodeling and this damage may account for the greater virulence. It is also possible that the enhanced expression of genes on chr 13 that we observed in the white strain created an imbalance in gene expression that directly or indirectly influenced the elaboration of known or unknown virulence factors. For example, it is likely that secreted factors contribute to pathology in the lung, and our observation that white, disomic strains have increased sensitivity to brefeldin A suggests an underlying perturbation of the secretory pathway.

The discovery of disomy in strain CBS7779 raises the question of whether this strain is particularly prone to genome instability, perhaps due to background defects in chromosome replication and segregation. The strain was initially selected for CGH analysis because Boekhout and van Belkum [41] reported that it had an unusually small genome (15 Mb versus 18-27 Mb for other strains). However, CGH did not reveal differences in content relative to the reference genome of strain H99 [18]. In the current study, the ease in detecting additional chromosome changes in our analysis of second-generation variants supports the possibility of general genome instability in CBS7779. Conversely, chromosome and ploidy changes may be more common than previously appreciated in species of *Cryptococcus*, as indicated by reports of extensive genome variation including karyotype variability and changes in clinical and environmental strains of *C. neoformans *and *C. gattii *in culture and in mammalian hosts [17,41-46]. The study of Fries et al. [17] is particularly informative because they found differences in karyotypes (rearrangements and different numbers of chromosomes) in sequential isolates of *C. neoformans *from AIDS patients. In addition, they passaged strains through mice and documented similar changes in karyotypes in cells recovered for three of the six isolates. The karyotype variations likely reflect translocations and segmental changes, and it is possible that stress conditions in mammalian hosts (e.g., oxidative, nitrosative and temperature stresses) promote chromosome variation. In our mouse passage experiments, the percentage of black colonies recovered from tissue was higher than in the starting inoculum, suggesting that they arose during infection. Although we are cautious about drawing conclusions about the frequency of copy number variation based solely on melanin as a surrogate indicator, our subsequent qPCR and CGH analyses revealed multiple chromosome changes in variants after passage in mice. More detailed studies with tagged chromosomes and fluctuation analysis will be needed to more closely examine the frequency of copy number change in animals and in response to stress.

More recently, Sionov et al. [27] analyzed fluconazole heteroresistance in the commonly studied *C. neoformans *strain H99 and found copy number changes for several chromosomes. In this case, selection for high-level resistance was correlated with disomy for chrs 1 and 4, a reduced growth rate and lower virulence. They also found a difference in virulence between a fluconazole resistant derivative H99 R64 and the parental H99 strain. Specifically, ~20% of the mice infected with the disomic strain died by the end of the experiment compared with ~50% inoculated with the wild-type strain. The appearance of disomic chromosomes in fluconazole-resistant isolates also occurs in the related species *C. gattii *[47,48]. In our study, we demonstrated that disomy could be detected in strain H99 by tagging chr 13 with a neomycin resistance marker and selecting on high levels of neomycin. However, some isolates showed the expected amplification of chr 13 and others had changes in different chromosomes. Together, these studies suggest that copy number variation is a common property of *C. neoformans*.

Genomic variation in *C. neoformans *extends beyond aneuploidy to include the occurrence of diploid strains arising from a-α and same-sex mating, endoreplication or clonal mating, as well as the formation of polyploid cells in infected animals [19,20,25,49]. For example, Lengeler et al. [19] examined 10 hybrid strains thought to arise from the fusion of serotype A and D mating partners, and they found that six were diploid and four were aneuploid with DNA content between 1N and 2N. We subsequently examined three of these AD hybrids by CGH and found that some chromosomes were preferentially retained from one parental genome or the other [18]. For example, all three strains retained chr 1 from the serotype A parent and lost the serotype D homologue. A survey of an additional 16 AD hybrid strains revealed that chr 1 from the A serotype genomes was preferentially retained in 11 of the isolates [18]. Recently, Lin et al. [20] surveyed 489 clinical and environmental strains of A serotype and found that 7.8% were diploid based on fluorescence flow cytometry. Thus diploid strains are relatively common, although in contrast, only six of the isolates appeared to be aneuploid. Characterization of laboratory-constructed diploids demonstrated that elevated ploidy has a minor negative influence on virulence in mice, and it is clear that diploids contribute to the disease spectrum because of the occurrence of these strains and mixed infections in AIDS patients [28,49]. Interestingly, Desnos-Ollivier et al., [28] showed that mixed infections with strains of different mating type, serotype, genotype and ploidy occur at a high frequency (18.4%) in patients; many of the isolates (8/23) in this study were diploid. Multilocus sequence typing indicated that some of the isolates may have arisen through diploidization (endoreplication). In our analysis, one strain from an AIDS patient (HC-6) appeared to be diploid with a copy number difference at chr 6. An isolate from a second patient (HC-4) also contained an elevated copy number for a segment of chr 9. Additional complexity comes from the discovery of tetraploid or octoploid cells that arise as a proportion of cryptococcal cells in the lungs of infected mice [25,26]. It is possible that disomy may result from the reduction in chromosome number as cells transition from the diploid or polyploid states to the haploid complement of chromosomes. Overall, the emerging view is that aneuploidy and changes in ploidy are common features of *C. neoformans*.

The occurrence of aneuploidy in *C. neoformans *is reminiscent of the situation in other fungal pathogens, and especially in the pathogenic yeast *Candida albicans *[11]. The diploid genome of this fungus exhibits a high level of plasticity, as strains with chromosomal rearrangements, loss of heterozygosity and aneuploidy are frequently observed. In addition, mating between diploid strains yields tetraploid strains that do not undergo meiosis but instead display random chromosome loss. As in *C. neoformans*, clinical isolates of *C. albicans *show considerable genomic diversity with the detection of translocations, chromosomal truncations and the formation of extra chromosomes. Elegant studies have shown that whole-chromosome or segmental aneuploidy can readily be detected by CGH and that this variation is associated with a growth advantage on L-sorbose or D-arabinose and with resistance to fluconazole [7,11-15,50-52]. Considerable genomic variation also occurs during growth in mammalian hosts and it has been found that aneuploidy influences virulence [6,8,9,11,53,54]. Changes during DNA transformation also are observed in *C. albicans *and our observations on the appearance of disomic strains during neomycin selection indicates that similar variation may occur in *C. neoformans *[55,56]. Chromosome variation is also a feature of the haploid yeast pathogen *Candida glabrata*. Specifically, Poláková et al. [57] examined the chromosome complements of 40 clinical isolates and found considerable variation including frequent examples of translocations, segmental duplications and the appearance of novel chromosomes. The sequences in regions of variation may influence interactions with the host and disease outcomes because they encode proteases, phospholipases and potential antifungal drug transporters. In fact, an isolate carrying a novel minichromosome showed increased tolerance to fluconazole.

## Conclusions

The genome variability documented here and in earlier work [17,18,27] could influence the virulence and persistence of *C. neoformans*, and therefore impact disease outcome. Cryptococcal meningoencephalitis is difficult to treat, particularly in AIDS patients because of the persistence of the fungus (especially in the central nervous system). Long-term antifungal drug therapy is sometimes necessary to prevent the recurrence of cryptococcosis in these patients [58]. Changes in ploidy and reversible chromosome copy number during infection likely mediate changes in gene expression and phenotype that could impact latency, chronic infection, antifungal drug susceptibility and the response to host defenses. Certainly aneuploidy is beneficial in the response to stress in other fungi, and both aneuploidy and polyploidy may provide mechanisms for cells to generate adaptive variability and deal with environmental challenges [13,15,59-63]. In general, it is important to understand the genetic flexibility of the pathogen as an aspect of efforts to control infection, and a number of important questions remain. These include whether propagation in culture versus the host environment preferentially results in chromosome variation and whether selective pressure in the host (oxidative, nitrosative and temperature stress, and nutrient limitation) influences chromosome instability. It will also be important to determine whether specific chromosomal changes and underlying alterations in the expression of virulence factors contributes to adaptation in different host tissues, to latency, and to resistance to host defense mechanisms. The answers to these questions will reveal the range of phenotypic changes determined by copy number change at each chromosome, and further establish relevance to disease.

## Methods

### Strains and growth conditions

The strains for this study are listed in Additional file 7 and were maintained on yeast extract, peptone, dextrose medium (YPD; Difco). To screen for variants of strain CBS7779, single colonies were grown in liquid YPD overnight at 30°C, and plated on L-DOPA medium ((0.5 mM 3,4-hydroxy-L-phenylalanine [L-DOPA], 1 mM MgSO_4 _· 7H2O, 22 mM · KH_2_PO_4_, 3 μM thiamine-HCl, 0.1% glucose, 0.1% L-asparagine, pH 5.6) at ~500 cells per plate. The plates were incubated at 30°C for 3 days and melanin production (darker or lighter colony pigmentation) was evaluated visually. Strains from the cerebral spinal fluid of AIDS patients were obtained by plating directly on YPD plates. To minimize the potential influence of prolonged culture on chromosome number, single colonies were picked and individually cultured in 50 ml YPD overnight prior to genomic DNA isolation. Growth in liquid yeast nitrogen base medium was monitored in a TECAN plate reader over 72 hours.

To insert a selectable marker on chr 13 of strain H99, a construct containing the neomycin resistance cassette was prepared by overlapping PCR and introduced by biolistic transformation [64]. The construct replaced a fragment of 31 base pairs in an intergenic region (chr 13: 316840-316870). The primer sequences for the construct are given in Additional file 11. Transformants were screened by colony PCR and four positive strains with the correct insertion of the neomycin resistant cassette (#s 32, 33, 34 and 35) were selected. A single colony of each tagged strain was grown in YPD overnight at 30°C and then plated on L-DOPA medium containing neomycin (2 mg/ml). After three days at 30°C, black and white colonies were collected, genomic DNA was isolated and qPCR and/or CGH analysis was employed to examine the copy number of chr 13 as described [18].

### Comparative Genome Hybridization

The genomic DNA for hybridization experiments was isolated as previously described [18]. The genomic sequence of H99 was retrieved from the *Cryptococcus neoformans var. grubii *H99 Sequencing Project, Broad Institute of Harvard and MIT (http://www.broadinstitute.org/). The assembled sequences were used by NimbleGen Systems, Inc. (Madison, WI) to design and manufacture the oligonucleotide genomic arrays. Because the focus of this study was on chromosome copy number changes, rather than the duplication/deletion of single genes or genome fragments, the design for the arrays was modified for our earlier design [18], with oligonucleotide probes that cover all 14 chromosomes of H99 tiled at an average interval spacing of 133 bp on one strand. The average length of each probe was 50 bp (range 45 to 85 bp) and the average Tm of the probes on each array was 76°C. The number of oligonucleotide probes for this new design for H99 was 134, 953. The probes for each array were designed to uniquely match a single sequence in the genome and highly repetitive centromeric regions and the rDNA repeat cluster were not included. The hybridization procedures, data acquisition and analysis were as previously described [18].

### Phenotypic assays

Melanin production was examined on solid L-DOPA medium. Capsule measurements, and stress- and drug-response assays were performed as previously described [32,65]. Briefly, exponentially growing cultures of test strains were washed, resuspended in H_2_O, and adjusted to 2 × 10^4 ^cells/μl. The cell suspensions were serially diluted 10-fold, and 5 μl of each dilution was spotted on YPD and/or YNB plates supplemented with or without 20 μg/ml or 40 μg/ml brefeldin A, 0.5 mg/ml monensin, or 5 μg/ml fluconazole. Plates were incubated for two to five days at 30°C or 37°C and photographed. To estimate the frequency of spontaneous emergence of color variants *in vitro*, ~3 × 10^4 ^cells of each strain were plated onto L-DOPA medium. After 72 hours of incubation at 30°C, dark colonies originating from a white parent (e.g., CBS7779-W1, -W2, or -W3), or white colonies from a black parent, were identified, and frequencies were estimated by dividing the number of variant colonies by the total CFU. A similar method was used to estimate the frequencies of changes in melanization *in vivo*; the percentages of colonies of the total fungal load in each organ with color variant to parental phenotype was assessed.

### Virulence assays and histopathology

The virulence of each strain was examined using female BALB/c mice (4 to 6 weeks old) from Charles River Laboratories (Ontario, Canada). Fungal cells were grown in 5 ml of YPD at 30°C overnight, washed twice with PBS (Invitrogen, Canada), and resuspended in PBS. The BALB/c mice, in groups of 10, were anesthetized intraperitoneally with ketamine (80 mg/kg of body weight) and xylazine (5.5 mg/kg) in PBS and suspended on a silk thread by the superior incisors. A suspension of 5 × 10^4 ^cells in 50 μl was slowly dripped into the nares of the anesthetized mice, and the mice were suspended for 10 minutes on the thread. The number of cells in each inoculum was confirmed by CFU determination after plating serial dilutions. The health status of the mice was monitored daily post-inoculation. Mice reaching the humane endpoint were euthanized by CO_2 _anoxia. All ten mice from each infection group were used at the endpoint to assess fungal loads in organs. Following euthanasia, the brain and the lungs were aseptically removed and immersed in 1 ml sterile PBS containing a metal bead. Organs were homogenized using an automated tissue homogenizer (Retsch, PA, USA). The samples were serially (1:10) diluted and 100 μl of 10^-4 ^and 10^-5 ^dilutions were plated and spread with 4 mm glass beads on L-DOPA medium containing 100 μg/ml chloramphenicol. After 3 days of incubation at 30°C, the L-DOPA plates were photographed using a Nikon Coolpix 990 camera. The images were opened with AlphaImager 3400, and the CFUs were digitally assessed by AlphaEase FC software (Alpha Innotech, San Leandro, CA). The number of color variants was tallied manually.

Female BALB/c mice (8 weeks old) from Charles River Laboratories (Ontario, Canada) were used in histopathology experiments to examine whether CBS7779-B4 and CBS7779-W2 cells elicited differential immune responses and/or exhibited different dissemination efficiency *in vivo*. Groups of nine mice were inoculated as described above, and on days 7, 14, and 21 post-infection, three mice from each group were euthanized by CO2 asphyxiation, and lungs and brains were immediately fixed in 10% neutral buffered formalin. Tissue embedding and sectioning (5 μm) were prepared by Wax-IT Histology (Vancouver, Canada) and stained with hematoxylin and eosin (H&E) or Mayer's mucicarmine (MM) to visualize the cryptococcal capsule. Lung slides were examined by light microscopy (AxioSkop 2 MOT, Carl Zeiss Ltd, Canada) in a blinded fashion and scored for degree of fungal infiltration, tissue damage and inflammation. Brain sections were examined by light microscopy for the presence and distribution of cryptococcal cells.

All experiments with animals were conducted according to guidelines set by the Canadian Council on Animal Care. The protocol (A08-0586) for the virulence assays with mice was approved by the University of British Columbia's Committee on Animal Care (Animal Welfare Assurance Number A5090-01). *C. neoformans *isolates from patients were obtained as part of the Database and Specimen Repository for Infectious Disease-Related Studies (reference number CR3_Pro00005314), as approved the Duke University Internal Review Board.

### Statistical analyses

Statistical analysis of survival differences was performed by Kaplan-Meier survival curves and log rank tests. An unpaired, two-tailed t-test was used to determine the differences in mean CFU per organ (i.e., brain and lung) between mice inoculated with different strains. A P-value of 0.05 or less was considered significant. All statistical analyses were carried out using GraphPad Prism version 4 for Windows (GraphPad Software, San Diego, CA).

### Quantitative real-time PCR

Quantitative real time PCR was used to confirm the copy number of chromosomes 4 and 13 in selected colonies as previously described [18]. Each reaction mixture contained 100 ng of template DNA, 0.25 μM of forward and reverse primers, and Power SYBR Green PCR Master Mix (Applied Biosystems). An ABI 7500 instrument (Applied Biosystems) was used for signal detection and data collection. Primer sequences for genes CNA04650 (actin), *SMG1 *(chr 4), 00_070 (chr 4) and CNN00820 (chr 13) were used for CCNV determination, as listed in Additional file 12 and as previously described [18].

### Flow cytometry

To examine the genomic profile of the original parental strains (CBS7779, CBS7779B4-6, CBS7779W1-3) and selected strains harvested post-infection, flow cytometry was performed as described [49], with slight modifications. Briefly, overnight cultures were centrifuged at 10,000 rpm for 1 minute, washed once with 1 mL of distilled water, and 1 × 10^7 ^cells were harvested and fixed in 70% ethanol overnight at 4°C. Subsequently, the ethanol was removed and the cells were washed with NS buffer (10 mM Tris-HCl [pH 7.6], 250 mM sucrose, 1 mM EDTA [pH 8.0], 1 mM MgCl_2_, 0.1 mM CaCl_2_, 0.1 mM ZnCl_2_), and stained with 10 μg/mL propidium iodide (Sigma) in 0.5 mL NS buffer containing 1 mg/mL RNase A (Roche) at 4°C overnight. The stained cells were then suspended in 50 mM Tris-HCl (pH 8.0) (without sonification). Propidium iodide fluorescence was analyzed using a BD FACSCalibur (BD Biosciences, Canada) on the FL3 channel. Graphs and histograms were generated using Flowjo v8.8.6 (Ashland, OR).

### RNA labeling and microarray analysis

The strains CBS7779-W2 and CBS7779-B4 were used for microarray analysis. Three independent biological replicates for each strain were grown in 25 ml of YPD overnight at 30°C. Cells were washed twice with water, re-suspended in L-DOPA medium and then incubated at 30°C for 6 h prior to RNA extraction. RNA was purified with the RNeasy kit (Qiagen) and treated with DNase (Qiagen) following the manufacturer's recommendations. The quality of the RNA was analyzed with an Agilent 2100 Bioanalyzer. cDNA synthesis, labeling with Cy3 or Cy5, and array hybridization to 70-mer microarrays (version 2.0, http://www.mogene.com were performed by Mogene (MO, USA). Dye-swap experiments were performed for all hybridizations. Modification of the gene list of microarray probes was described previously [66]. Single 60-mer oligonucleotide probes were designed for each of the ORFs and were duplicated on the microarray to provide an estimate of intra array variance. Of the 7,302 ORFs designated by the Broad Institute, 100 ORFs were eliminated because the sequences were of low complexity. The probes covered 6,932 of the final annotated genes. Agilent Technologies fabricated the arrays.

After ANOVA normalization, genes showing log_2_-transformed fold changes (*P *< 0.05) were selected for further analyses. To classify genes by their functions, a web-based program, CateGOrizer (http://www.animalgenome.org/bioinfo/tools/catego/), was used [67]. The heatmap was generated using the "heatmap.2" routine in the package gplots after the gene expression data were normalized using R-package, by limma with options of 'minimum' background correction, 'Lowess' within-array normalization, and 'quantile' between-array normalization as recommended [68].

## Abbreviations

CGH: comparative genome hybridization; chr: chromosome; PCR: polymerase chain reaction

## Competing interests

The authors declare that they have no competing interests.

## Authors' contributions

GH, IL, JC, APL, TGM, JRP, TB and JWK conceived and designed the research. GH, JW, JC, and IL performed the experiments. RA designed the microarray for the genome of strain H99, and GH, JW, IL JC and WHJ analyzed the data. JC, RA, TB, APL, TGM and JRP contributed reagents/materials and analysis tools. GH, JW, JC, IL, AL, TM, JP, TB and JK wrote the paper. All authors read and approved the final manuscript.

## Supplementary Material

Additional file 1 **Genes up-regulated in the black strain B4 (Table S1)**. A microarray comparison of the transcriptomes of the black B4 isolate and the white W2 isolate identified genes with greater than two-fold elevated transcript levels in the B4 isolate. Click here for file

Additional file 2 **Genes up-regulated in the white strain W2 (Table S2)**. A microarray comparison of the transcriptomes of the black B4 isolate and the white W2 isolate identified genes with greater than two-fold elevated transcript levels in the W2 isolate. Click here for file

Additional file 3 **Comparison of gene expression for a second-generation black strain (Figure S1)**. Gene expression was compared for the CBS7779-B4 strain and the "second generation" black strain CBS7779-W2BA by microarray analysis. Strain CBS7779-W2BA was obtained from the white strain CBS7779-W2 (Figure 1). Two arrays were employed and two biological repeats were performed to examine transcript levels. Columns 1 and 2 each represent a microarray experiment and each row represents the expression of a gene on the array arranged by its chromosomal position. The relative expression levels are represented by color as shown in the bar. Click here for file

Additional file 4 **CGH analysis of additional strains with chromosome copy number variation (Figure S2)**. Passage of the white variant CBS7779-W2 (disomic for chr 13) in culture and analysis of black or white isolates by CGH revealed changes at additional chromosomes. The black variant W2-BB showed copy number increase for a segment of chr 12, and the white isolate W2-WB gained a segmental changes for chr 12 and chr 14. The black variant strain CBS7779-B1 was confirmed to be monosomic by CGH and additional variants were identified in culture (Figure 6). In addition, white variants of strain B1 were subsequently screened for black variants (B1-WC-B1, disomic for chr 13; B1-WC-B4, monosomic for all chromosomes). Click here for file

Additional file 5 **CGH analysis of additional strains obtained from mice (Figure S3)**. Passage of the CBS7779 variants W2, W3 and B6 in mice lead to variation at chr 4 and chr 13. As described in the text and presented in Figure 6, white and black strains collected from the lungs and brains of mice infected with CBS7779 variants were compared to the reference genome of strain H99 by CGH. Click here for file

Additional file 6 **Phenotypic characterization of variants of the laboratory strain H99 (Figure S4)**. (A) The screen for variants with reduced melanin production is shown for tagged strains on L-DOPA medium containing neomycin. This procedure yielded the strains analyzed in Figure 7. (B) The capsule size and cell morphology of the variants were examined with india ink staining. This size bar is 10 μm. (C) Summary of the phenotypes of each variant. Click here for file

Additional file 7 **Strain list (Table S3)**. The strains employed in the study are listed in Table S3. Click here for file

Additional file 8 **CGH analysis of 18 clinical and environmental strains (Figure S5)**. The strains were analyzed along with strains A5-35-17 and JP1086 (Figure 8) using the array for the reference strain H99. Click here for file

Additional file 9 **CGH analysis of isolates from HIV/AIDS patients (Figure S6**). Three isolated colonies were tested for patients HC-2, HC-3 and HC-5, and a representative colony is shown for the three colonies from patient HC-6. The analysis of another HC-6 colony is shown in Figure 8 (all three colonies had the same chromosome complement). Click here for file

Additional file 10 **Analysis of three HC-6 isolates by fluorescence-activated flow cytometry (Figure S7)**. The ploidy of the three isolates from patient HC-6 were examined and the haploid strain H99 and the diploid strain KW5 were included as controls. Click here for file

Additional file 11 **Primer list for strain construction (Table S4)**. The primers employed to insert a selectable marker on chr 13 of strain H99 are listed in Table S4. Click here for file

Additional file 12 **Primer list for qPCR (Table S5)**. The primers employed for quantitative real time PCR to confirm the copy number of chromosomes 4 and 13 are listed in Table S5. Click here for file
