# Supplementary figures and images for: Variation in chromosome copy number influences the virulence of Cryptococcus neoformans and occurs in isolates from AIDS patients
Source: BMC Genomics. 2011 Oct 27;12:526. doi: 10.1186/1471-2164-12-526 (PMC3221739; doi:10.1186/1471-2164-12-526)

Figure S1

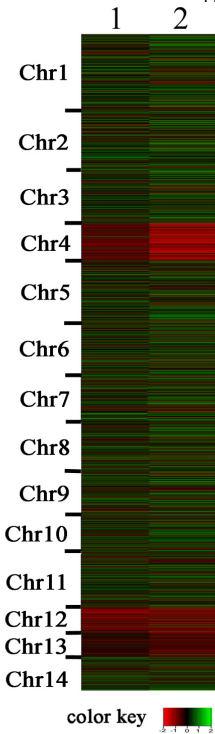

Supplement: Additional file 3 — Comparison of gene expression for a second-generation black strain (Figure S1). Gene expression was compared for the CBS7779-B4 strain and the "second generation" black strain CBS7779-W2BA by microarray analysis. Strain CBS7779-W2BA was obtained from the white strain CBS7779-W2 (Figure 1). Two arrays were employed and two biological repeats were performed to examine transcript levels. Columns 1 and 2 each represent a microarray experiment and each row represents the expression of a gene on the array arranged by its chromosomal position. The relative expression levels are represented by color as shown in the bar. [file 1471-2164-12-526-S3.PDF]

Figure S2

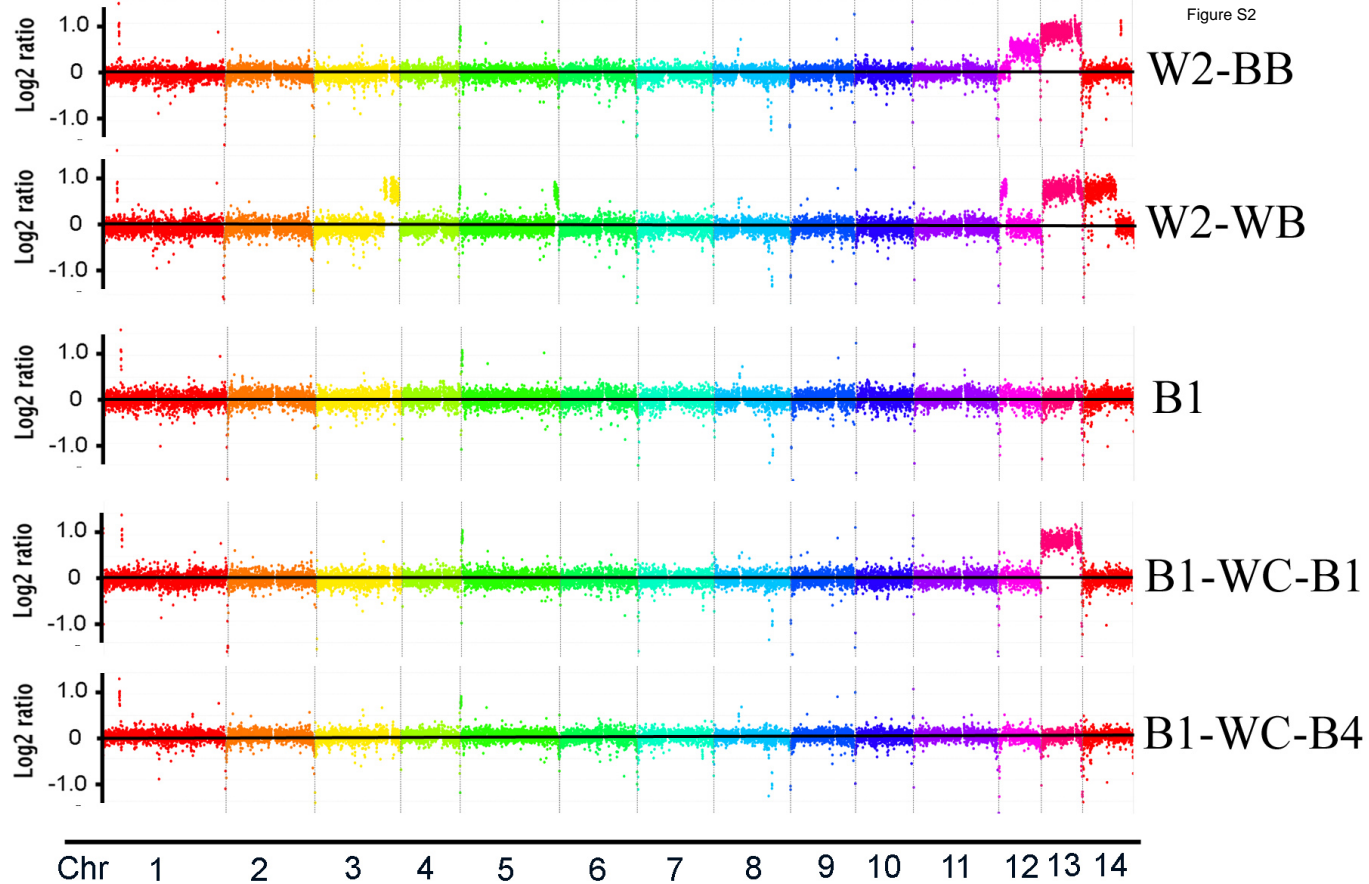

Supplement: Additional file 4 — CGH analysis of additional strains with chromosome copy number variation (Figure S2). Passage of the white variant CBS7779-W2 (disomic for chr 13) in culture and analysis of black or white isolates by CGH revealed changes at additional chromosomes. The black variant W2-BB showed copy number increase for a segment of chr 12, and the white isolate W2-WB gained a segmental changes for chr 12 and chr 14. The black variant strain CBS7779-B1 was confirmed to be monosomic by CGH and additional variants were identified in culture (Figure 6). In addition, white variants of strain B1 were subsequently screened for black variants (B1-WC-B1, disomic for chr 13; B1-WC-B4, monosomic for all chromosomes). [file 1471-2164-12-526-S4.PDF]

Figure S3

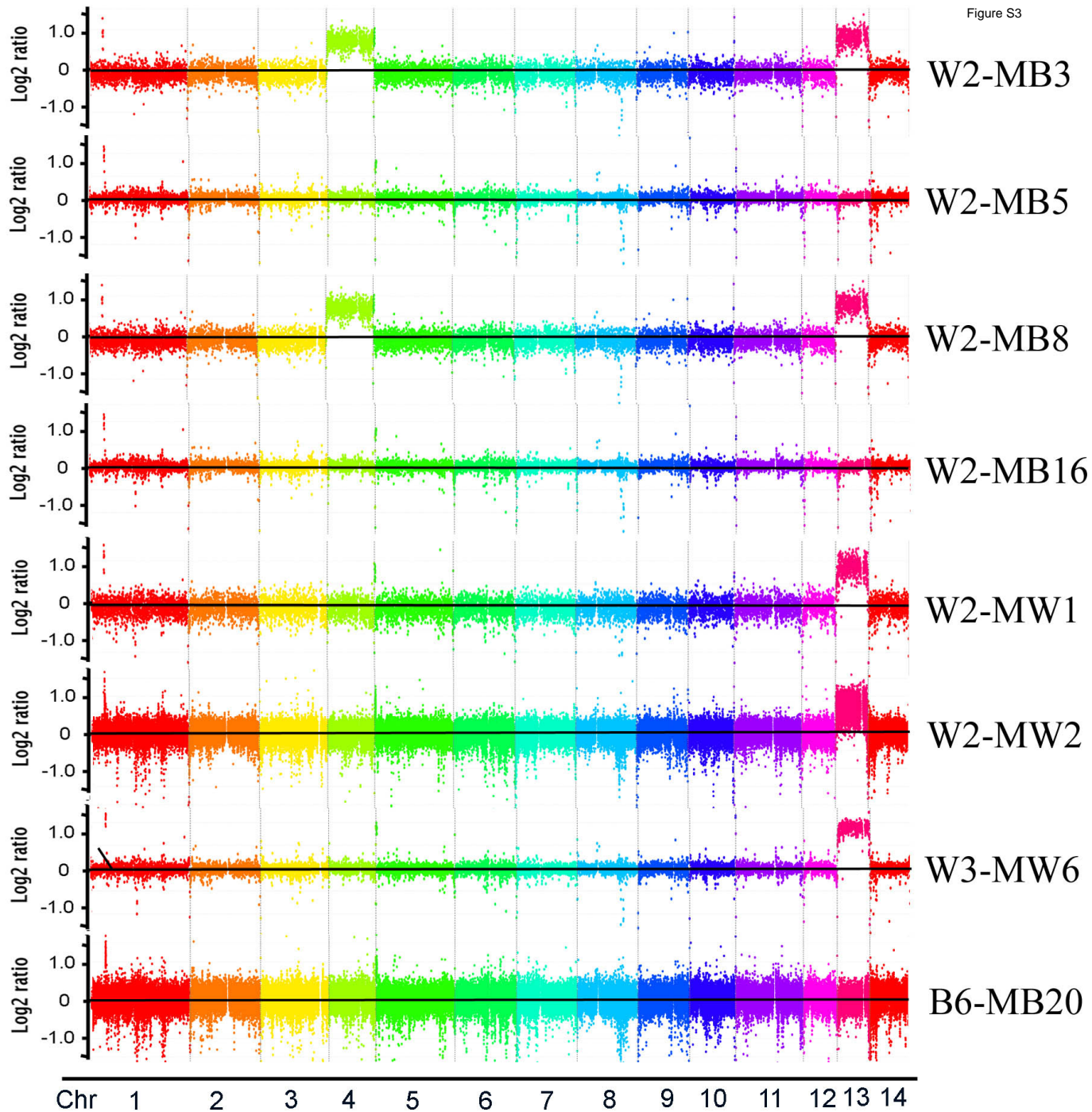

Supplement: Additional file 5 — CGH analysis of additional strains obtained from mice (Figure S3). Passage of the CBS7779 variants W2, W3 and B6 in mice lead to variation at chr 4 and chr 13. As described in the text and presented in Figure 6, white and black strains collected from the lungs and brains of mice infected with CBS7779 variants were compared to the reference genome of strain H99 by CGH. [file 1471-2164-12-526-S5.PDF]

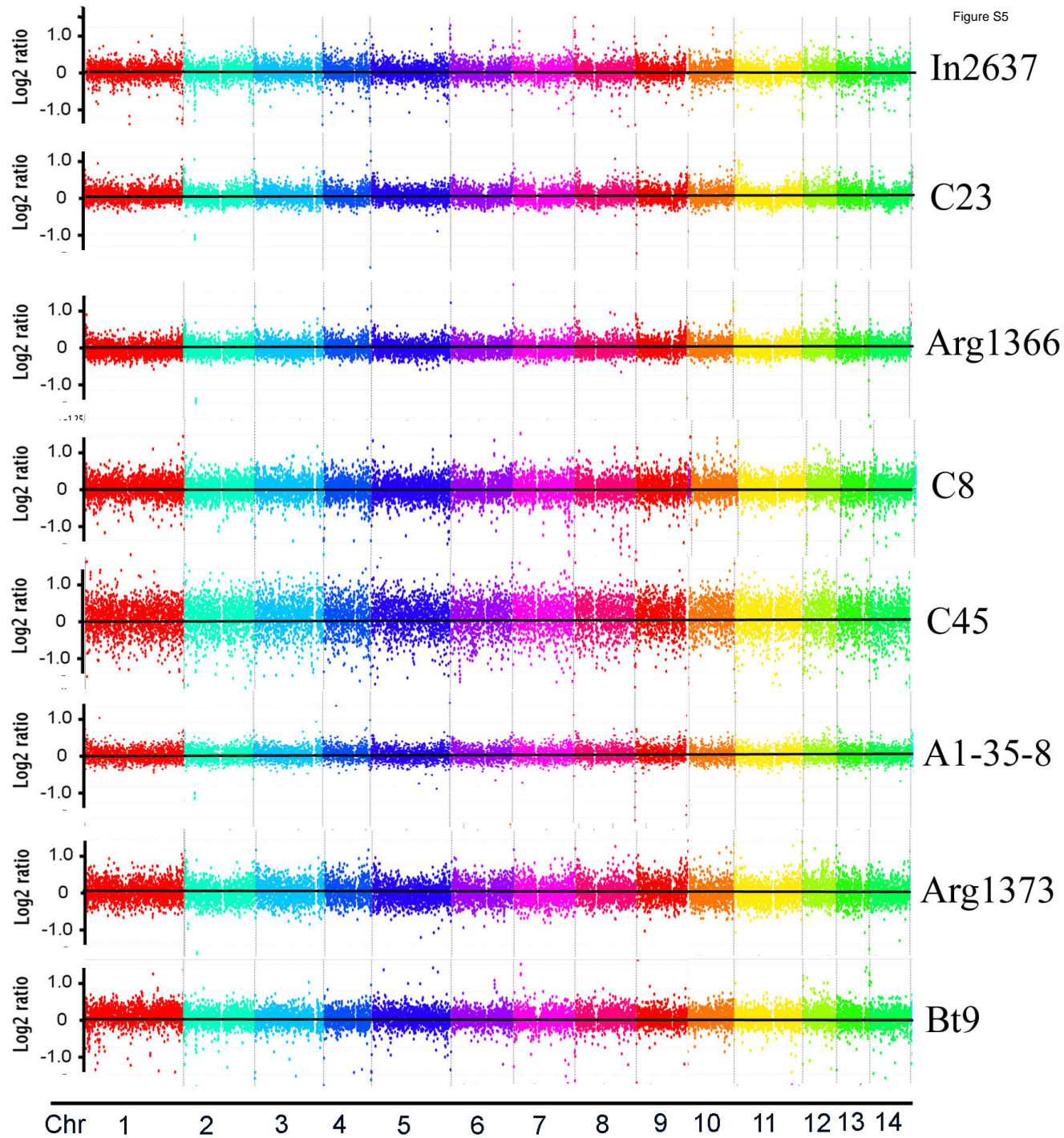

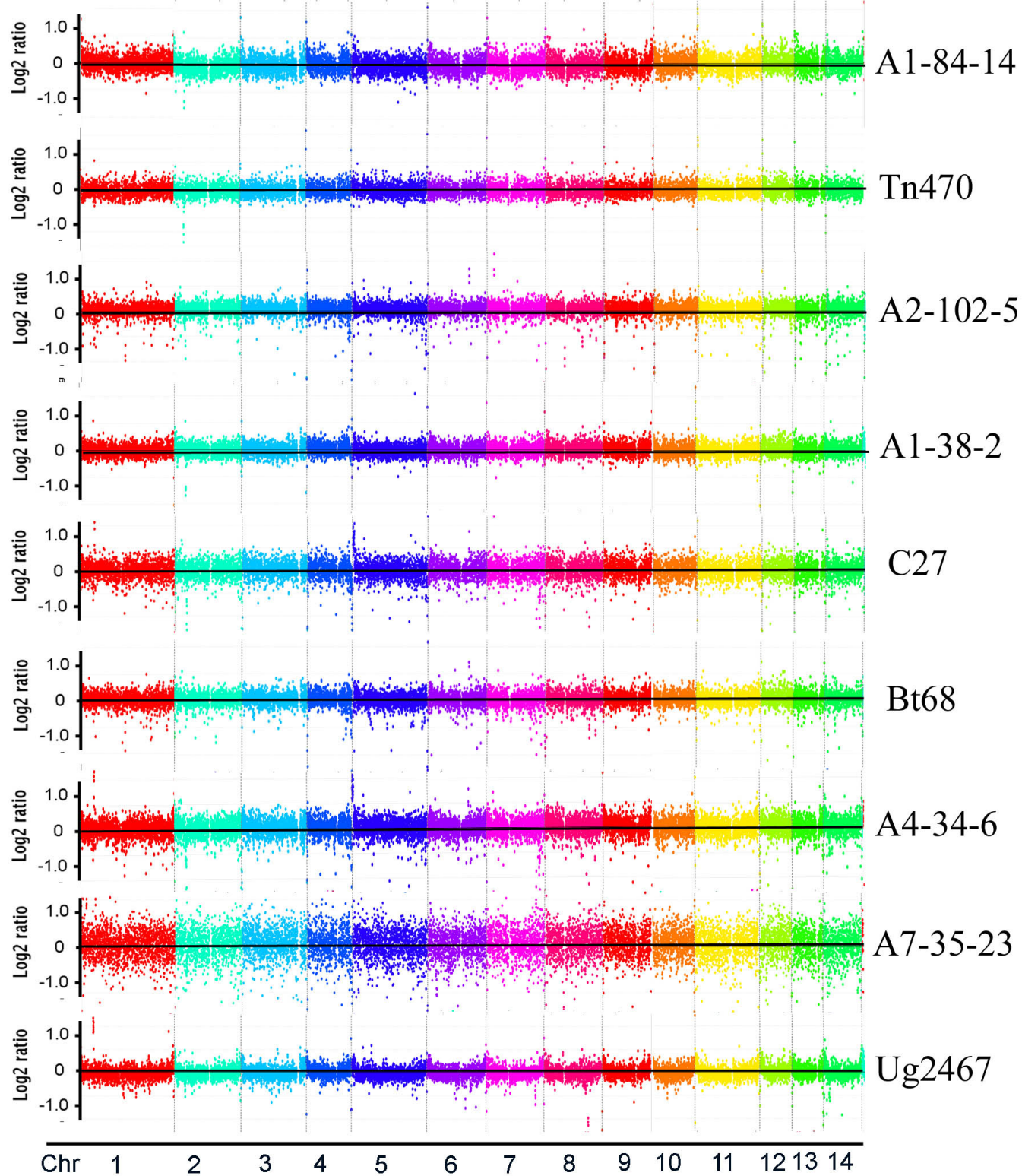

Supplement: Additional file 8 — CGH analysis of 18 clinical and environmental strains (Figure S5). The strains were analyzed along with strains A5-35-17 and JP1086 (Figure 8) using the array for the reference strain H99. [file 1471-2164-12-526-S8.PDF]

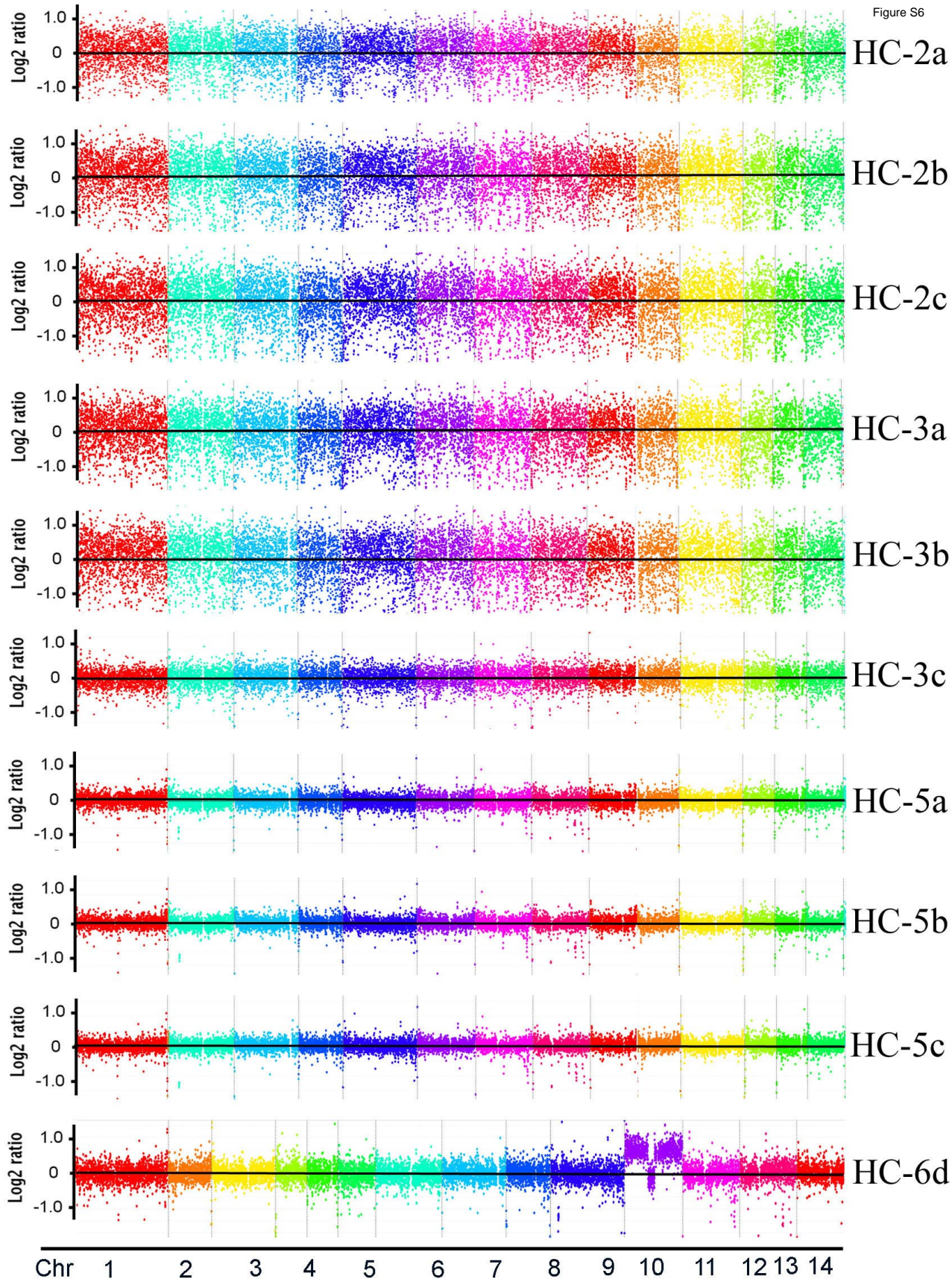

Supplement: Additional file 9 — CGH analysis of isolates from HIV/AIDS patients (Figure S6). Three isolated colonies were tested for patients HC-2, HC-3 and HC-5, and a representative colony is shown for the three colonies from patient HC-6. The analysis of another HC-6 colony is shown in Figure 8 (all three colonies had the same chromosome complement). [file 1471-2164-12-526-S9.PDF]

Figure S7

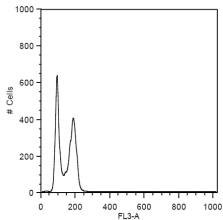

H99

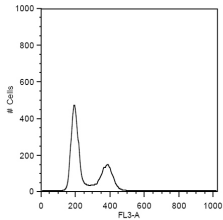

KW5

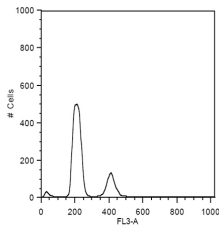

HC-6a

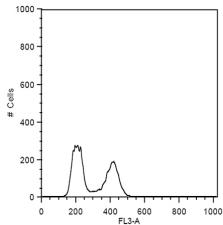

HC-6b

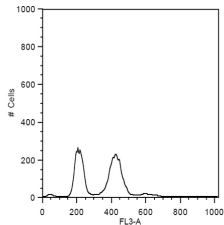

HC-6c

Supplement: Additional file 10 — Analysis of three HC-6 isolates by fluorescence-activated flow cytometry (Figure S7). The ploidy of the three isolates from patient HC-6 were examined and the haploid strain H99 and the diploid strain KW5 were included as controls. [file 1471-2164-12-526-S10.PDF]
