# Supplementary material for: Variation in chromosome copy number influences the virulence of Cryptococcus neoformans and occurs in isolates from AIDS patients
Source: BMC Genomics. 2011 Oct 27;12:526. doi: 10.1186/1471-2164-12-526 (PMC3221739; doi:10.1186/1471-2164-12-526)
Supplement: Additional file 6 — Phenotypic characterization of variants of the laboratory strain H99 (Figure S4). (A) The screen for variants with reduced melanin production is shown for tagged strains on L-DOPA medium containing neomycin. This procedure yielded the strains analyzed in Figure 7. (B) The capsule size and cell morphology of the variants were examined with india ink staining. This size bar is 10 μm. (C) Summary of the phenotypes of each variant. [file 1471-2164-12-526-S6.PDF]

Figure S4

A

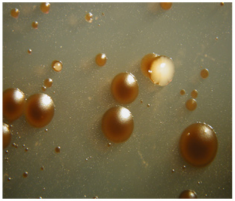

B

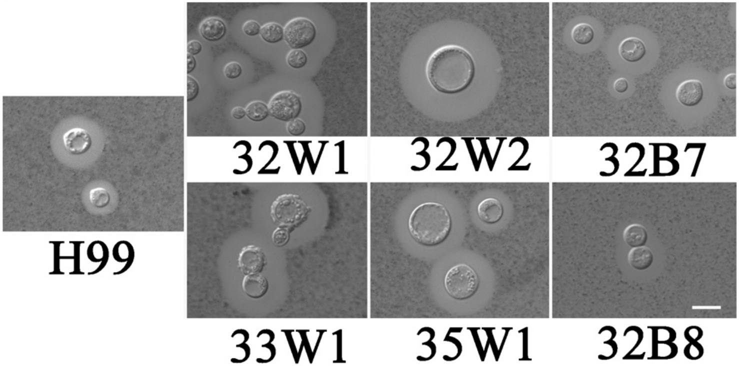

C

Chromosome variation observed in H99 isolates tagged on chromosome 13 with a neomycin resistance marker and selected for resistance to neomycin

H99 variant	Melanin	Chromosome	Other phenotypes
32W1	White	Chr 11, Chr 14	Giant cells
35W1	White	Chr 13, Chr 14	Giant cells, large capsule
34W2	White	none	
33W1	White	none	Large capsule
32B8	Black	none	
32B7	Black	Chr 13 partial 9, 14	
