# Supplementary material for: Variation in chromosome copy number influences the virulence of Cryptococcus neoformans and occurs in isolates from AIDS patients
Source: BMC Genomics. 2011 Oct 27;12:526. doi: 10.1186/1471-2164-12-526 (PMC3221739; doi:10.1186/1471-2164-12-526)
Supplement: Additional file 7 — Strain list (Table S3). The strains employed in the study are listed in Table S3. [file 1471-2164-12-526-S7.DOC]

| **Table S3. Strains** | | | | |
| --- | --- | --- | --- | --- |
| **Strains** | **Molecular subtype/Genotype** |  | **CGH** | **Source/Reference** |
| H99 | VNI |  |  | J. Heitman |
| CBS7779 | VNI |  | Yes | T. Boekhout |
| WM626 | VNI |  | Yes | W. Meyer |
|  |  |  |  |  |
| CBS7779 – W1 | VNI |  | Yes | This study |
| CBS7779 – W2 | VNI |  | Yes | This study |
| CBS7779 – W3 | VNI |  | Yes | This study |
| CBS7779 – W4 | VNI |  |  | This study |
| CBS7779 – W5 | VNI |  |  | This study |
| CBS7779 – W6 | VNI |  |  | This study |
| CBS7779 – W7 | VNI |  |  | This study |
| CBS7779 – W8 | VNI |  |  | This study |
| CBS7779 – W9 | VNI |  |  | This study |
| CBS7779 – W10 | VNI |  |  | This study |
| CBS7779 – W11 | VNI |  |  | This study |
| CBS7779 – W12 | VNI |  |  | This study |
|  |  |  |  |  |
| CBS7779 - B1 | VNI |  | Yes | This study |
| CBS7779 – B2 | VNI |  |  | This study |
| CBS7779 – B3 | VNI |  |  | This study |
| CBS7779 – B4 | VNI |  | Yes | This study |
| CBS7779 – B5 | VNI |  | Yes | This study |
| CBS7779 – B6 | VNI |  | Yes | This study |
| CBS7779 – B7 | VNI |  |  | This study |
| CBS7779 – B8 | VNI |  |  | This study |
| CBS7779 – B9 | VNI |  |  | This study |
| CBS7779 – B10 | VNI |  |  | This study |
| CBS7779 – B11 | VNI |  |  | This study |
| CBS7779 – B12 | VNI |  |  | This study |
|  |  |  |  |  |
| W2-BA | VNI |  | Yes | This study |
| W2-BB | VNI |  | Yes | This study |
|  |  |  |  |  |
| W1-BA | VNI |  |  | This study |
| W2-WB | VNI |  | Yes | This study |
|  |  |  |  |  |
| W3-BB | VNI |  | Yes | This study |
| W3-BC | VNI |  | Yes | This study |
|  |  |  |  |  |
| B1-WA | VNI |  | Yes | This study |
| B1-WB | VNI |  | Yes | This study |
| B1-WC | VNI |  | Yes | This study |
|  |  |  |  |  |
| B1-WCB1 | VNI |  | Yes | This study |
| B1-WCB4 | VNI |  | Yes | This study |
|  |  |  |  |  |
| B6-MW10 | VNI |  | Yes | This study |
| B6-MB20 | VNI |  | Yes | This study |
|  |  |  |  |  |
| W2-MW1 | VNI |  | Yes | This study |
| W2-MW2 | VNI |  | Yes | This study |
| W2-MB3 | VNI |  | Yes | This study |
| W2-MB4 | VNI |  | Yes | This study |
| W2-MB5 | VNI |  | Yes | This study |
| W2-MB7 | VNI |  | Yes | This study |
| W2-MB8 | VNI |  | Yes | This study |
|  |  |  |  |  |
| W3-MW6 | VNI |  | Yes | This study |
| W3-MB16 | VNI |  | Yes | This study |
| W3-MB13 | VNI |  | Yes | This study |
|  |  |  |  |  |
| HC-2a, b, c | Unknown |  | Yes | T. Bicanic/J. Perfect |
| HC-3a, b, c | Unknown |  | Yes | T. Bicanic/J. Perfect |
| HC-4a, b | Unknown |  | Yes | T. Bicanic/J. Perfect |
| HC-5 a, b, c | Unknown |  | Yes | T. Bicanic/J. Perfect |
| HC-6, c, d | Unknown |  | Yes | T. Bicanic/J. Perfect |
|  |  |  |  |  |
| RTC23 -1, 2, 3 | Unknown |  | Yes | T. Bicanic/J. Perfect |
| RTC31-1, mix | Unknown |  | Yes | T. Bicanic/J. Perfect |
|  |  |  |  |  |
| RCT17A,B,C | Unknown |  | Yes | T. Bicanic/J. Perfect |
| RCT50-1,2,3 | Unknown |  | Yes | T. Bicanic/J. Perfect |
| RCT52-1,2,3 | Unknown |  | Yes | T. Bicanic/J. Perfect |
| RCT55 – day1 | Unknown |  | Yes | T. Bicanic/J. Perfect |
| RCT55 – day 4, 1,2,3 | Unknown |  | Yes | T. Bicanic/J. Perfect |
| RCT60 -1,2,3 | Unknown |  | Yes | T. Bicanic/J. Perfect |
|  |  |  |  |  |
| JP1086 | VNI, A5/M5 |  | Yes | [23] |
| Arg1373 | VNI, A1/M1 |  | Yes | [23] |
| Arg1366 | VNI, A1/M1 |  | Yes | [23] |
| Ug2467 | VNI, A3/M3 |  | Yes | [23] |
| In2637 | VNI, A3/M3a |  | Yes | [23] |
| Tn470 | VNI, A1/M1 |  | Yes | [23] |
| Bt9 | VNI, gen26(A1)M43 |  | Yes | [23] |
| Bt68 | VNI, gen33/M17 |  | Yes | [23] |
| C8 | VNI, A5/M5 |  | Yes | [23] |
| A1-35-8 | VNI, A1/M1 |  | Yes | [24] |
| A1-38-2 | VNI, A1/M1 |  | Yes | [24] |
| A1-84-14 | VNI, A1/M1 |  | Yes | [24] |
| A7-35-23 | VNII |  |  | [24] |
| A2-102-5 | VNI, A2/M2 |  | Yes | [24] |
| A4-34-6 | VNI, A4/M4 |  | Yes | [24] |
| A5-35-17 | VNI, A5/M5 |  | Yes | [24] |
| C23 | VNI, A1/M1 |  | Yes | [24] |
| C27 | VNI, A4/M4 |  | Yes | [24] |
| C45 | VNII A6/M7c |  | Yes | [24] |
| 1-25 | VNI, A5/M5 |  |  | T. Mitchell |
| 19-6 | VNI, A5/M5 |  |  | T. Mitchell |
| 38-3 | VNI, A5/M5 |  |  | T. Mitchell |
| 102-1 | VNI, A5/M5 |  |  | T. Mitchell |
| D16-11 | VNI, A5/M5 |  |  | T. Mitchell |
| J0278-1 | VNI, A5/M5 |  |  | T. Mitchell |
| RSA730 | VNI, A5/M5 |  |  | T. Mitchell |
| RSA731 | VNI, A5/M5 |  | Yes | T. Mitchell |
|  |  |  |  |  |
| H99 - 35W1 | VNI |  | Yes | This study |
| H99 - 34W2 | VNI |  | Yes | This study |
| H99 - 32B8 | VNI |  | Yes | This study |
| H99 - 32B7 | VNI |  | Yes | This study |
| H99 - 33W1 | VNI |  | Yes | This study |
| H99- 32W1 | VNI |  | Yes | This study |
